# *In vitro* DNA Inversions Mediated by the PsrA Site-Specific Tyrosine Recombinase of *Streptococcus pneumoniae*

**DOI:** 10.3389/fmolb.2020.00043

**Published:** 2020-03-19

**Authors:** Jingwen Li, Juanjuan Wang, Sofía Ruiz-Cruz, Manuel Espinosa, Jing-Ren Zhang, Alicia Bravo

**Affiliations:** ^1^Department of Basic Medical Science, Center for Infectious Disease Research, School of Medicine, Tsinghua University, Beijing, China; ^2^Centro de Investigaciones Biológicas Margarita Salas, Consejo Superior de Investigaciones Científicas, Madrid, Spain

**Keywords:** tyrosine recombinase PsrA, *Streptococcus pneumoniae*, type I restriction-modification *cod* locus, site-specific DNA inversions, inverted repeats

## Abstract

Site-specific recombination is a DNA breaking and reconstructing process that plays important roles in various cellular pathways for both prokaryotes and eukaryotes. This process requires a site-specific recombinase and direct or inverted repeats. Some tyrosine site-specific recombinases catalyze DNA inversions and regulate subpopulation diversity and phase variation in many bacterial species. In *Streptococcus pneumoniae*, the PsrA tyrosine recombinase was shown to control DNA inversions in the three DNA methyltransferase *hsdS* genes of the type I restriction-modification *cod* locus. Such DNA inversions are mediated by three inverted repeats (IR1, IR2, and IR3). In this work, we purified an untagged form of the PsrA protein and studied its DNA-binding and catalytic features. Gel retardation assays showed that PsrA binds to linear and supercoiled DNAs, containing or not inverted repeats. Nevertheless, DNase I footprinting assays showed that, on linear DNAs, PsrA has a preference for sites that include an IR1 sequence (IR1.1 or IR1.2) and its boundary sequences. Furthermore, on supercoiled DNAs, PsrA was able to generate DNA inversions between specific inverted repeats (IR1, IR2, and IR3), which supports its ability to locate specific target sites. Unlike other site-specific recombinases, PsrA showed reliance on magnesium ions for efficient catalysis of IR1-mediated DNA inversions. We discuss that PsrA might find its specific binding sites on the bacterial genome by a mechanism that involves transitory non-specific interactions between protein and DNA.

## Introduction

Site-specific recombination (SSR) is a DNA breaking and reconstructing process widely distributed in both prokaryotes and eukaryotes, in which a specialized enzyme catalyzes reciprocal strand exchange at specific target sites. According to this fundamental definition, two critical elements participate in the process, namely a site-specific recombinase and a pair of inversely or directly repeated sequences (Grindley et al., 2006; Rajeev et al., 2009). Based on the amino acid residue involved in the catalysis, site-specific recombinases have been classified into two families: tyrosine (Tyr) and serine (Ser) recombinases, both of them employing the C-terminal OH group of the active residue to perform the nucleophilic attack for DNA sequence exchange (Grindley et al., 2006). In addition to the difference in the catalytic residues, the catalytic mechanisms of Tyr- and Ser-recombinases are different. In the former, after the nucleophilic attack, the catalytic Tyr residue remains covalently linked to the 3′-end of the DNA strand, generating a 3′-phospho-tyrosyl bond and leaving a free 5′-OH group. Besides, Tyr-recombinases cleave just one DNA strand every time, and only after the cleaved strand is rejoined at the crossover site forming a unique holiday junction (HJ) intermediate, the uncleaved strand can be cut by its partner within the same dimer. In contrast, Ser-recombinases form a 5′-phosphor-serine bond generating free 3′-OH intermediates. Further, the enzyme always cleaves both strands at a time and does not generate the HJ intermediate. Unlike general homologous recombination event that requires large-size homologous DNA segments, DNA replication and high-energy cofactors, the specific repeated sequences recognized by site-specific recombinases are usually 20–40 bp in length, and no DNA synthesis or energy factors are required (Grindley et al., 2006).

In bacteria, SSR is an efficient and feasible way to generate genetic rearrangements, leading to regulation of subpopulation diversity, or phase variation and cellular adaption to various environments at physiological and phenotypic level (Darmon and Leach, 2014). As one of the typical forms of SSR, DNA inversion is an important regulatory mechanism to introduce genetic and phenotypic variation in a clonal population, also referred to intercellular heterogeneity, which is well-characterized in several pathogenic bacterial species (Darmon and Leach, 2014). In particular, promoter inversion is one of the most commonly used strategies to regulate surface antigen expression, as in the cases of the *fim* switch of *Escherichia coli* (Abraham et al., 1985; Olsen and Klemm, 1994) and the flagellar switch in *Salmonella typhimurium* (Zieg and Simon, 1980; Johnson et al., 1986). In addition, genetic rearrangement to switch gene contents is also a practical strategy widely adopted in bacteria, such as the VlsE lipoprotein variation in *Borrellia burgdorferi* (Zhang et al., 1997; Norris, 2014) and the V-1 surface antigen variation in *Mycoplasma pulmonis* (Bhugra et al., 1995; Shen et al., 2000).

**Figure 1:**
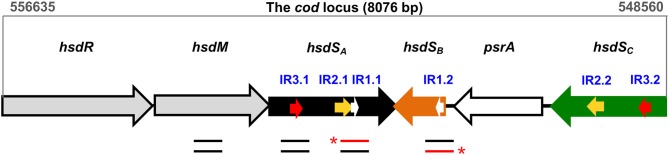
Genetic organization of the *cod* locus. The *cod* locus of the pneumococcal ST556 genome (coordinates 556635–548560) consists of six genes: *hsdR* (restriction enzyme), *hsdM* (DNA methyltransferase), three *hsdS* homologs (*hsdS*_*A*_, *hsdS*_*B*_, *hsdS*_*C*_), and *psrA* (tyrosine recombinase). Three pairs of inverted repeats, which mediate site-specific DNA inversions, are indicated with white arrows (IR1.1/IR1.2), yellow arrows (IR2.1/IR2.2), and red arrows (IR3.1/IR3.2), respectively. The location of DNA fragments used in EMSA and/or DNase I footprinting assays is indicated with lines below genes. The red lines indicate the DNA strands that were radiolabeled at the 5′-end (asterisks).

Studies from our (Feng et al., 2014; Li et al., 2016; Li and Zhang, 2019) and other laboratories (Manso et al., 2014; De Ste Croix et al., 2017; de Ste Croix et al., 2019) revealed that DNA inversions in the *hsdS* genes of the *cod* locus of *Streptococcus pneumoniae* (the pneumococcus) lead to diversification of genomic DNA methylation patterns, and phenotypic variation in colony opacity. As a type I restriction-modification (RM) system, the *cod* locus is composed of six genes, including *hsdR* (restriction enzyme), *hsdM* (DNA methyltransferase), three target recognition subunit *hsdS* homologs, and *psrA* (site-specific tyrosine recombinase) (Figure 1). Among the three *hsdS* genes, only the *hsdS*_*A*_ gene, which is co-transcribed with *hsdR* and *hsdM*, is functional to recognize the specific methylation motif, whereas *hsdS*_*B*_ and *hsdS*_*C*_ genes are serving as DNA sources for sequence switching with *hsdS*_*A*_ by inversions (see also Figure 1). Three pairs of inverted repeats (IRs), termed 15-bp IR1, 298-bp IR2, and 85-bp IR3, were identified to drive sequence exchange between *hsdS*_*A*_ and *hsdS*_*B*_/*hsdS*_*C*_ (Li et al., 2016). Further investigation focusing on the molecular mechanisms of site-specific *hsdS* inversions in the *cod* locus revealed that pneumococci employ multiple pathways to catalyze *hsdS* inversions between the three inverted repeats, including dominant PsrA-driven inversions and less frequent inversions that don't require PsrA (Li et al., 2019). Particularly, the Tyr-recombinase PsrA plays an essential role in catalyzing all three inversion reactions mediated by IR1, IR2, and IR3. While IR1 inversions are strictly dependent on PsrA catalysis, IR2- and IR3-inversions are dramatically reduced in *psrA* mutant strains. Sequence replacement of the IR1 repeat and its boundary sequences with irrelevant sequences demonstrated that efficient catalysis by PsrA *in vivo* depends on the sequence specificity of the 15-bp IR1 repeat and its immediately upstream sequence, which are supposed to be the target sequence where PsrA performs a nucleophilic attack or interacts directly. In addition, PsrA-mediated IR2- and IR3-inversions rely on the presence of IR1-like sequences in the IR2 and IR3 repeats. However, the precise interaction of PsrA with the IR1 repeat and the molecular mechanism of its catalytic activity remain to be elucidated.

In the present work, we have purified an untagged form of the pneumococcal PsrA protein and studied its DNA-binding and catalytic features. Our results suggest that PsrA might locate its preferred target sites (specific interactions) by a mechanism that involves transient binding at random DNA sites (non-specific interactions). Different from other Tyr-recombinases, protein PsrA exhibited a marked dependence on Mg^2+^ cations for efficient catalysis *in vitro*.

## Results

### Purification of PsrA

**Figure 2:**
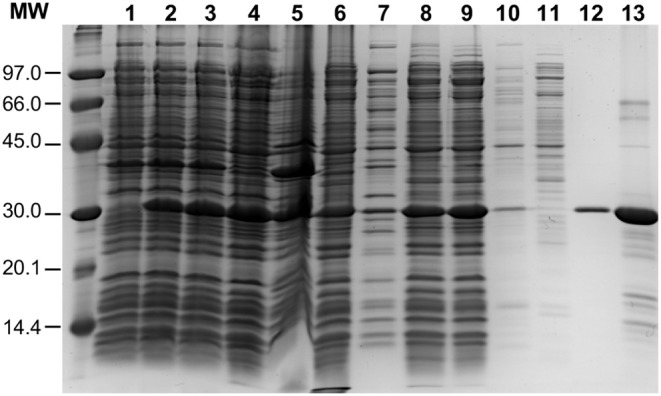
Purification of PsrA. PsrA was overproduced using *E. coli* BL21 (DE3)/pTH12647 cells. Lane 1: whole-cell extract before adding IPTG. Lane 2: whole-cell extract from cells treated with IPTG (25 min). Lane 3: whole-cell extract from cells treated with IPTG (25 min) and then with rifampicin (60 min). The latter whole-cell extract (French Pressure Cell) was centrifuged: supernatant (lane 4; cleared cell lysate) and pellet (lane 5, cell debris). Precipitation of nucleic acids with PEI: supernatant (lane 6) and pellet (lane 7). Lane 8: Proteins recovered by precipitation with ammonium sulfate; this fraction was further dialyzed against buffer S containing 300 mM NaCl and centrifuged: supernatant (lane 9) and pellet (lane 10). The protein preparation shown in lane 9 was loaded onto a heparin affinity column: flow-through fraction (lane 11) and PsrA-containing fraction (lane 12). Lane 13: PsrA preparation after heparin chromatography. Proteins were analyzed by SDS-PAGE (12%). Gels were stained with Coomassie Blue. Molecular weight (MW) standards (in kDa) were run in the same gel (LMW Marker, GE Healthcare).

To overproduce an untagged version of the pneumococcal PsrA site-specific recombinase, the *psrA* gene was inserted into the *E. coli* inducible expression vector pET24b, which is based on the phi10 promoter recognized by the T7 RNA polymerase. The recombinant plasmid (pTH12647) was then introduced into the *E. coli* BL21 (DE3) strain that carries the T7 RNA polymerase-encoding gene fused to the *lacUV5* promoter (Studier and Moffatt, 1986). This strain carries also a chromosomal copy of the *lacI* repressor gene. Thus, expression of the T7 RNA polymerase-encoding gene, and consequently expression of the *psrA* gene, is induced when isopropyl β-D-1-thiogalactopyranoside (IPTG) is added to the bacterial culture (see Materials and Methods) (Figure 2, lane 2). The procedure used for large-scale purification of PsrA involved essentially the following steps (Figure 2): (i) precipitation of nucleic acids with polyethyleneimine (PEI) (0.2%) in the presence of NaCl (300 mM). The ionic strength at which PEI precipitation was done was high enough to keep PsrA from binding to DNA (lane 6); (ii) after PEI precipitation, proteins in the supernatant (including PsrA) were precipitated with 70% saturated ammonium sulfate; and (iii) chromatography on a heparin column using a linear gradient of NaCl (300–800 mM) for protein elution. Protein PsrA was eluted at 675 mM NaCl. Purified PsrA was analyzed by SDS-polyacrylamide (PAA) (12%) gel electrophoresis. The PsrA preparation obtained after heparin chromatography was about 90% pure (lane 13). The 260/280 absorbance ratio for the purified protein was 0.52. PsrA migrated slightly above the 30 kDa reference band, which is consistent with the molecular weight of the PsrA monomer (31.2 kDa) calculated from the predicted amino acid sequence.

### Binding of PsrA to Linear Double-Stranded DNAs

**Figure 3:**
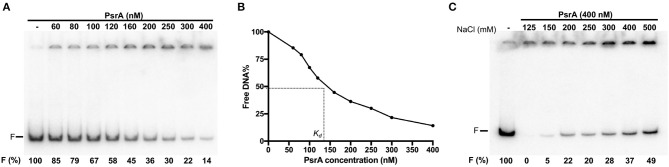
Formation of IR1.2-PsrA complexes in the absence of competitor DNA. **(A)** Binding of PsrA (60–400 nM) to the ^32^P-labeled IR1.2 DNA (4 nM). Reactions were loaded onto a native gel (6% PAA). DNA was visualized using a Fujifilm Image Analyzer FLA-3000. The band corresponding to free DNA (F) and the percentage of free DNA in each reaction (F%) are indicated. **(B)** Free DNA from the experiment shown in A was quantified using the Quantity One software (Bio-Rad), and the percentage of free DNA was plotted against the concentration of PsrA. *K*_d_: dissociation constant. **(C)** Effect of increasing concentrations of NaCl on the binding of PsrA (400 nM) to the ^32^P-labeled IR1.2 DNA (2 nM).

To analyse the DNA-binding properties of PsrA, we performed EMSA experiments using different linear double-stranded DNAs (dsDNAs). First, we used a radioactively labeled 265-bp DNA fragment (coordinates 550883-550619 of the pneumococcal ST556 genome). This fragment (here named IR1.2 DNA) contains the IR1.2 repeat (Figure 1). The labeled IR1.2 DNA (4 nM) was incubated with increasing concentrations of PsrA (60–400 nM) in the absence of competitor DNA. Free DNA and bound DNA were separated by native PAA (6%) gel electrophoresis (Figure 3A). As the protein concentration was increased, free IR1.2 DNA disappeared gradually and IR1.2-PsrA complexes, which did not enter the gel, were generated. The PsrA concentration required to bind half the DNA was determined by measuring the decrease in free DNA rather than the increase in complexes, which gives an indication of the approximate magnitude of the dissociation constant, *K*_d_. Such a concentration was about 145 nM (Figure 3B). However, this value would underestimate the affinity of PsrA for its primary binding site if multiple protein units bind to the same DNA molecule. IR1.2-PsrA complexes unable to enter the gel were also observed when the binding reactions were analyzed by electrophoresis on agarose (1%) gels (Figure S1). By EMSA, we also analyzed the effect of NaCl on the binding reaction. PsrA (400 nM) was incubated with the labeled IR1.2 DNA in the presence of different concentrations of NaCl (125–500 mM) (Figure 3C). As the salt concentration was increased, the amount of free IR1.2 DNA increased, indicating that the formation of IR1.2-PsrA complexes is impaired at high NaCl concentration.

**Figure 4:**
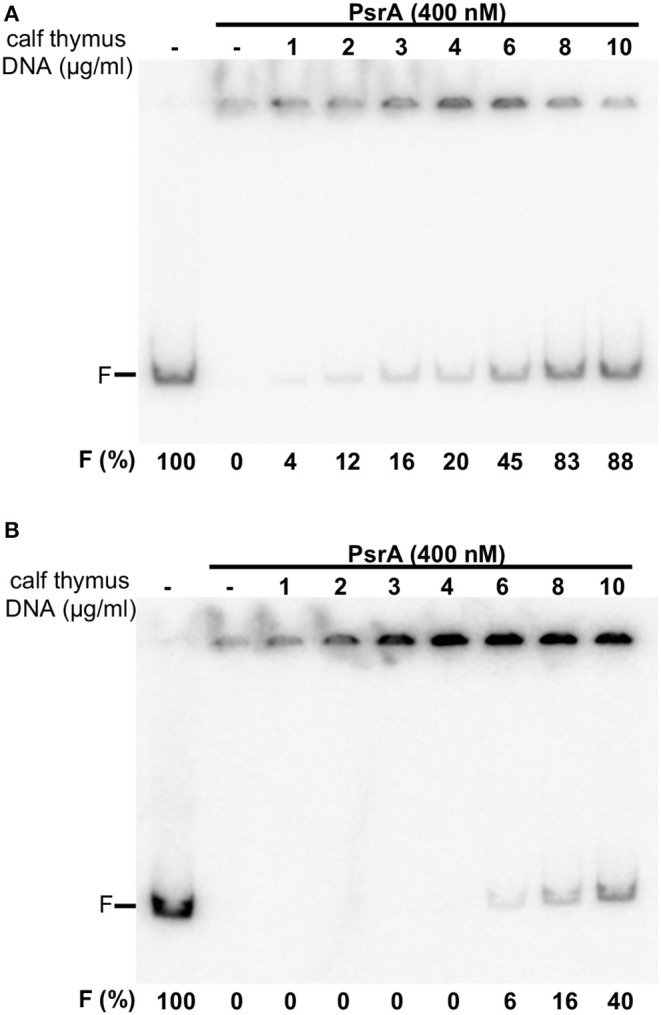
Formation of IR1.2-PsrA complexes in the presence of competitor DNA. **(A)** Effect of increasing concentrations of calf thymus DNA on the binding of PsrA (400 nM) to the ^32^P-labeled IR1.2 DNA (4 nM). Reactions were loaded onto a native gel (6% PAA). The band corresponding to free DNA (F) and the percentage of free DNA in each reaction (F%) are indicated **(B)** Dissociation of IR1.2-PsrA complexes. The indicated concentration of non-labeled calf thymus DNA was added to pre-formed IR1.2-PsrA complexes.

Next, the labeled IR1.2 DNA (4 nM, 0.65 μg/ml) was incubated with PsrA (400 nM) in the presence of different concentrations of non-labeled competitor calf thymus DNA (1–10 μg/ml) (Figure 4A). In the absence of competitor DNA, no free IR1.2 DNA was visualized. As the concentration of competitor DNA was increased, the amount of free IR1.2 DNA increased. However, only 20% of the IR1.2 DNA (0.13 μg/ml) moved as free DNA in the presence of 4 μg/ml of competitor DNA, which indicated that PsrA binds preferentially to the IR1.2 DNA. We also performed dissociation experiments using non-labeled competitor calf thymus DNA (Figure 4B). In this case, PsrA was first incubated with the labeled IR1.2 DNA for 20 min at room temperature. Under these conditions, free IR1.2 DNA molecules were not detected (formation of IR1.2-PsrA complexes). Then, different concentrations of competitor DNA were added to the reactions. After 5 min, reaction mixtures were loaded onto a native PAA (6%) gel. At 8 μg/ml of competitor DNA, only 16% of the IR1.2 DNA (0.10 μg/ml) moved as free DNA. Thus, PsrA dissociated from the IR1.2-PsrA complexes at high concentrations of competitor DNA, which can be taken as an indication of complex stability. IR1.2-PsrA interactions were also disrupted when heparin was used as a competitor (Figure S2).

**Figure 5:**
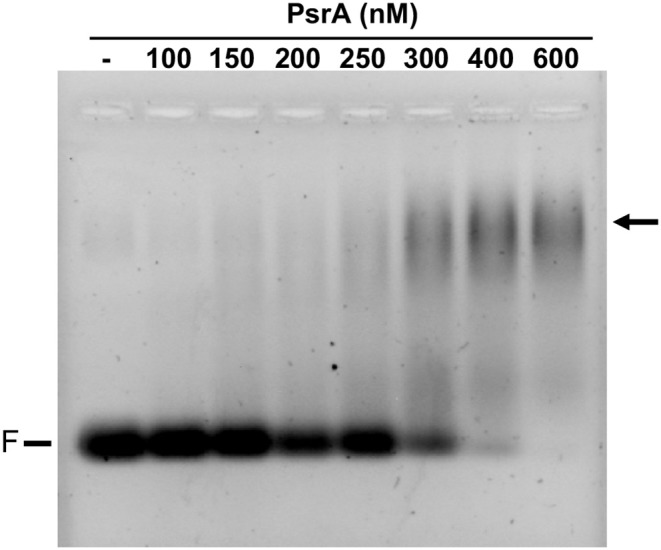
Binding of PsrA to a 26-bp DNA fragment. The indicated concentration of PsrA was mixed with 50 nM of DNA. Binding reactions were analyzed by agarose (2%) gel electrophoresis. DNA was stained with GelRed (Biotium). F: free DNA. The position of DNA-PsrA complexes is indicated with an arrow.

Further EMSA experiments were performed using other linear dsDNA fragments from the pneumococcal ST556 genome: (i) a 265-bp DNA fragment that contains the IR1.1 repeat (here named IR1.1 DNA, coordinates 551690-551954; Figure 1); (ii) a 260-bp DNA fragment that contains the IR3.1 repeat (coordinates 552497-552756; Figure 1); and (iii) a 265-bp DNA fragment (coordinates 553471-553735) amplified from the *hsdM* gene, which lacks IRs. In all cases, DNA-PsrA complexes that did not enter the gel were generated (Figure S3). Moreover, we analyzed the binding of PsrA to a 26-bp dsDNA obtained by annealing of complementary oligonucleotides. These oligonucleotides do not contain IRs (Solano-Collado et al., 2013). In this case, DNA-PsrA complexes moving slower than free DNA were visualized by agarose (2%) gel electrophoresis (Figure 5). However, such complexes did not enter native PAA (8%) gels (data not shown). Thus, large DNA-PsrA complexes were also generated with the 26-bp dsDNA.

Taken all the above results together, we can conclude that gel retardation assays showed that PsrA binds to linear dsDNAs in a non-sequence-specific manner generating higher-order complexes. Similar results were obtained when supercoiled plasmid DNAs, containing or not IR1 repeats, were used as target DNAs (Figure S4).

### DNase I Footprinting Analysis of IR1-PsrA Complexes

**Figure 6:**
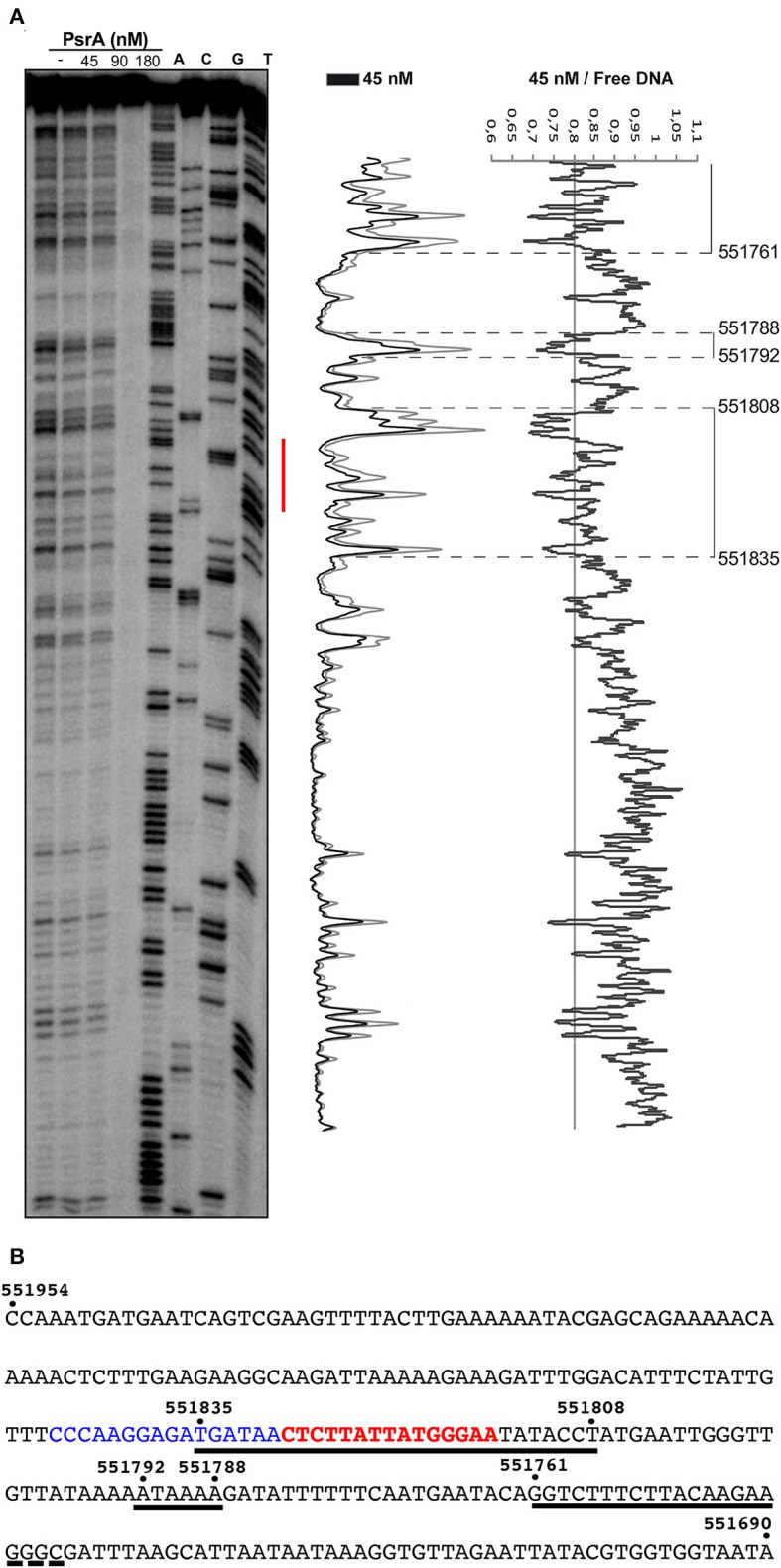
DNase I footprints of complexes formed by PsrA on the IR1.1 DNA. **(A)** ^32^P-labeled IR1.1 DNA (6 nM) was incubated with the indicated concentrations of PsrA (formation of complexes) and then digested with DNase I. Dideoxy-mediated chain termination sequencing reactions were run in the same gel (lanes A, C, G, T). The red bar indicates the position of the IR1.1 repeat. Densitometer scans corresponding to DNA with PsrA (45 nM; black line) and DNA without PsrA (gray line) are shown. The ratio of intensities between both scans is also shown. **(B)** Nucleotide sequence of the IR1.1 DNA fragment (coordinates 551954-551690 of the ST556 genome). Regions protected against DNase I digestion in the presence of PsrA (45 nM) are indicated with black lines. The IR1.1 repeat sequence (in red) and the upstream adjacent sequence (in blue) are required for PsrA-mediated IR1 inversions *in vivo* (Li et al., 2019).

**Figure 7:**
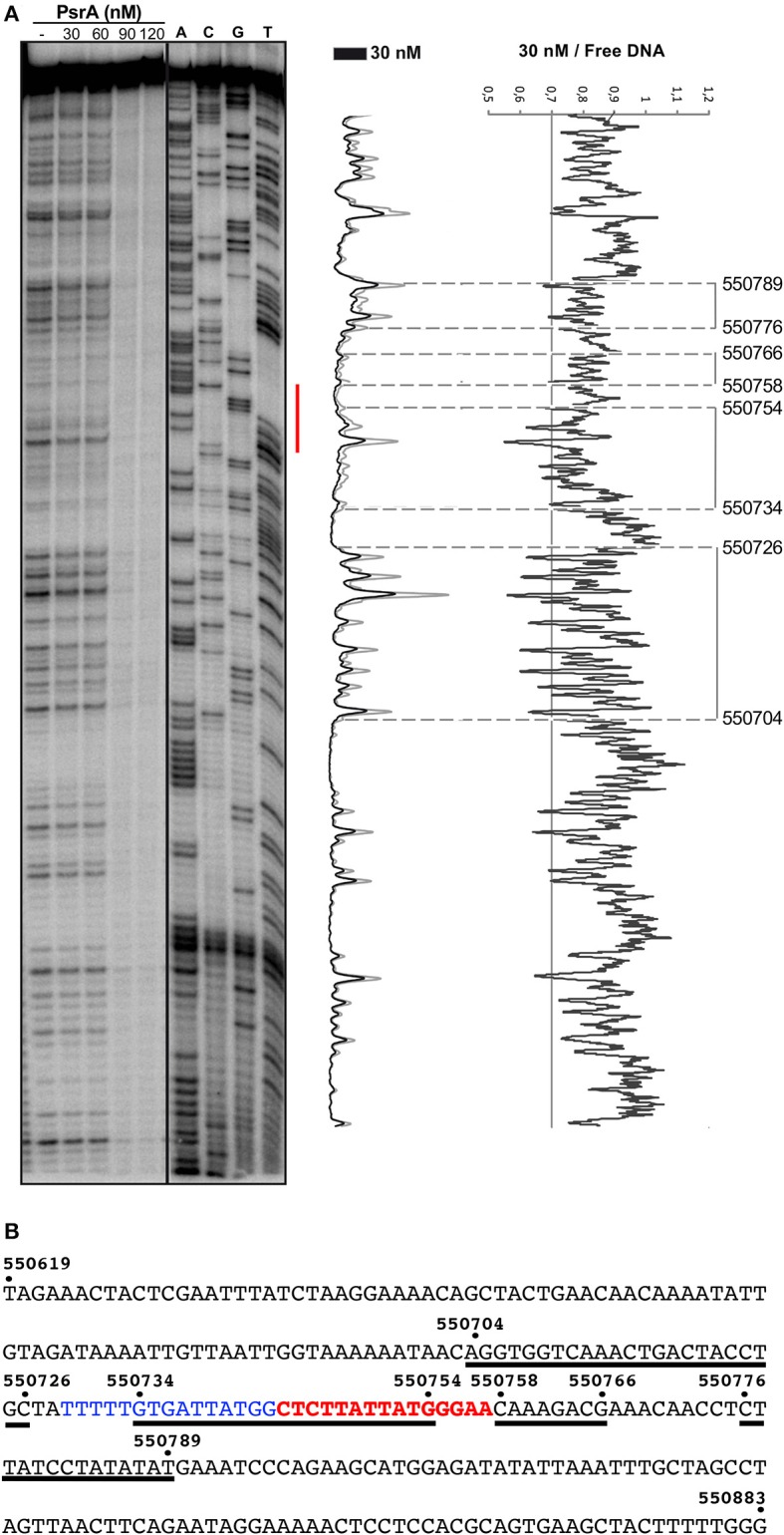
DNase I footprints of complexes formed by PsrA on the IR1.2 DNA. **(A)** ^32^P-labeled IR1.2 DNA (4 nM) was incubated with the indicated concentrations of PsrA (formation of complexes) and then digested with DNase I. Dideoxy-mediated chain termination sequencing reactions were run in the same gel (lanes A, C, G, T). The red bar indicates the position of the IR1.2 repeat. Densitometer scans corresponding to DNA with PsrA (30 nM; black line) and DNA without PsrA (gray line) are shown. The ratio of intensities between both scans is also shown. **(B)** Nucleotide sequence of the IR1.2 DNA fragment (coordinates 550619–550883 of the ST556 genome). Regions protected against DNase I digestion in the presence of PsrA (30 nM) are indicated with black lines. The IR1.2 repeat sequence (in red) and the upstream adjacent sequence (in blue) are required for PsrA-mediated IR1 inversions *in vivo* (Li et al., 2019).

*In vivo* studies showed that PsrA is necessary and sufficient for IR1-mediated inversions. Moreover, PsrA requires both the 15-bp inverted repeats (IR1.1 and IR1.2) and the immediately upstream sequences of both repeats (Li et al., 2019). Here, we analyzed the interaction of PsrA with the IR1.1 and IR1.2 DNA fragments by DNase I footprinting experiments (Figures 6, 7). The 265-bp IR1.1 DNA (see Figure 1) was radioactively labeled at the 5′-end of the coding strand (coordinate 551954; Figure 6). Labeled DNA (6 nM) was incubated with increasing concentrations of PsrA. At 45 nM, changes in DNase I sensitivity (diminished cleavages) were observed from coordinate 551835 to 551808, from 551792 to 551788, and from position 551761 onwards. Thus, the region protected against DNase I digestion includes the IR1.1 repeat (15-bp), the bordering regions (6-bp) and sequences located downstream of IR1.1 (Figure 6). When PsrA concentration was increased to 180 nM, DNase I cleavage was highly reduced, indicating the formation of higher-order IR1.1-PsrA complexes, in which the DNA was not anymore accessible to the DNase I attack.

The 265-bp IR1.2 DNA fragment was labeled at the 5′-end of the coding strand (coordinate 550619) (see Figure 1). Labeled DNA (4 nM) was incubated with increasing concentrations of PsrA. At 30 nM, diminished cleavages were observed within the region spanning coordinates 550704 and 550789 (Figure 7). Thus, PsrA recognizes a region that includes the IR1.2 repeat and its adjacent sequences. At higher PsrA concentrations (90 nM), IR1.2-PsrA complexes insensitive to DNase I cleavage were generated (Figure 7).

### PsrA Catalyzes the Formation of IR1-Mediated Inversions *in vitro*

**Figure 8:**
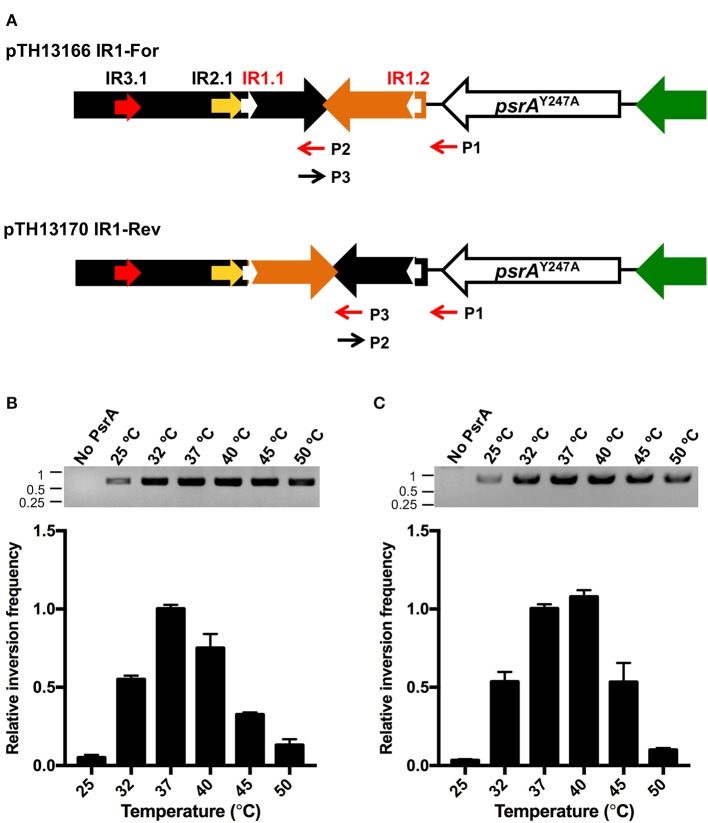
PsrA generates IR1 inversions *in vitro*. **(A)** Relevant features of the plasmids used to analyse PsrA-mediated inversions between IR1.1 and IR1.2. Primers used to detect IR1 inversions are indicated with red arrows. Primers P2 and P3 have complementary sequences. **(B)** Effect of temperature on the formation of IR1 inversions (from “For” to “Rev”) using plasmid pTH13166. PCR assays (agarose gel) and qPCR assays (bar graph) were carried out using the P1/P2 primers. The position of DNA molecular weight markers (in kb) is indicated on the left of the gel. **(C)** Effect of temperature on the formation of IR1 inversions (from “Rev” to “For”) using plasmid pTH13170. PCR assays (agarose gel) and qPCR assays (bar graph) were performed using the P1/P3 primers.

We further investigated whether PsrA was able to generate IR1 inversions *in vitro*. To this end, we constructed the recombinant plasmids pTH13166 (IR1-For) and pTH13170 (IR1-Rev), harboring S1 and S2 configurations of the *cod* locus, respectively (Figure 8A). In both plasmids, the IR2.2 and IR3.2 repeats were removed to avoid the impact of IR2- and IR3-mediated inversions. Plasmid pTH13166 was used to examine IR1 inversions from “For state” to “Rev state,” whereas the employment of plasmid pTH13170 allowed us to examine IR1 inversions from “Rev state” to “For state.” To detect the occurrence of IR1 inversions, both PCR and qPCR assays were carried out using primers P1/P2 for pTH13166 and P1/P3 for pTH13170 (Figure 8A). PCR results were analyzed by agarose gel electrophoresis (upper part in Figures 8B,C). The qPCR data were used to calculate the relative inversion frequency (bar graphs in Figures 8B,C). In a first approach, PsrA (125 nM) was mixed with plasmid DNA (1 nM) in the presence of NaCl (125 mM) and MgCl_2_ (5 mM). Reaction mixtures were incubated at different temperatures for 1 h (Figures 8B,C). IR1 inversions were detected in both plasmids only in the presence of PsrA. Thus, we could demonstrate that protein PsrA generates IR1 inversions *in vitro*. The optimum reaction temperature was 37°C in plasmid pTH13166 (Figure 8B) and 37–40°C in plasmid pTH13170 (Figure 8C), corresponding to the optimal temperature for pneumococci to grow.

Next, we determined the relative frequency of IR1 inversions in the presence of PsrA as a function of the incubation time. In both plasmids (pTH13166 and pTH13170), the maximum frequency of inversions was observed after 2 h incubation (bar graphs in Figure S5). Moreover, we examined the effect of pH and NaCl concentration on the frequency of PsrA-mediated IR1 inversions. The highest inversion frequencies were observed at a pH value of 7.6–8.0 and 150 mM NaCl in plasmid pTH13166 (Figures S6A,C) and at a pH value of 8.0 and 125 mM NaCl in plasmid pTH13170 (Figures S6B,D).

### PsrA Relies on Mg^2+^ to Achieve Efficient IR1 Inversion Reactions

**Figure 9:**
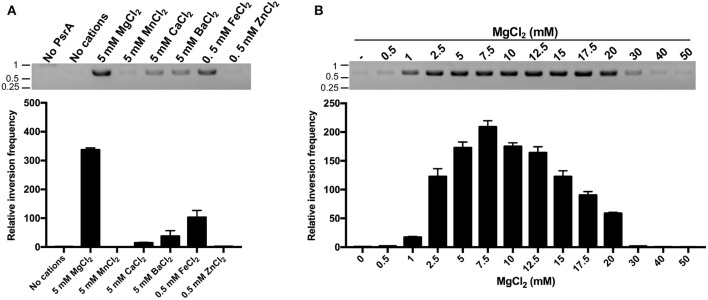
Mg^2+^ enhances the frequency of PsrA-mediated IR1 inversions. **(A)** Relative frequency of PsrA-mediated IR1 inversions in the presence of different divalent cations (bar graph). Plasmid pTH13166 (1 nM) was incubated with PsrA (125 nM) at 37°C for 1 h. IR1 inversions were detected by PCR assays (agarose gel) and by qPCR assays (bar graph) using the P1/P2 primers (see Figure 8A). The position of DNA molecular weight markers (in kb) is indicated on the left of the gel. **(B)** Relative frequency of PsrA-mediated IR1 inversions using plasmid pTH13166 and the indicated concentrations of MgCl_2_ (bar graph). IR1 inversions were detected by PCR assays (agarose gel) and by qPCR assays (bar graph).

Current understanding of the mechanisms of site-specific recombination, based on *in vitro* studies, proposes that site-specific recombinases do not require high-energy factors and/or divalent cations for catalysis (Mack et al., 1992; Grindley et al., 2006; Fan, 2012). However, here we found that this does not seem to be the case for the pneumococcal tyrosine recombinase PsrA. First, we analyzed the effect of various divalent cations (Mg^2+^, Mn^2+^, Ca^2+^, Ba^2+^, Fe^2+^, and Zn^2+^) on the efficiency of the PsrA-mediated IR1 inversion reactions. Compared to the reaction without divalent cations, the highest frequency of DNA inversions was observed in the presence of 5 mM MgCl_2_ (~335-fold; plasmid pTH13166) (Figure 9A). Similar results were obtained with plasmid pTH13170 (Figure S7). The frequency of IR1 inversions increased also to some extent in the presence of 5 mM CaCl_2_, 5 mM BaCl_2_, and 0.5 mM FeCl_2_ (14-, 37-, and 102-fold in pTH13166, and 2-, 3-, and 13-fold in pTH13170) (Figure 9A and Figure S7A). We also examined the effect of various concentrations of MgCl_2_ (0.5–50 mM) on the efficiency of the PsrA-mediated IR1 inversion reaction. The maximum frequency of inversions was observed at 7.5 mM of MgCl_2_ in both plasmids (Figure 9B and Figure S7B). By EMSA experiments, we found that neither MgCl_2_ (5 mM) nor FeCl_2_ (0.5 mM) influences the binding of PsrA to DNA fragments that contain the IR1.2 repeat (Figure S8). Thus, Mg^2+^ might play a significant role in PsrA catalysis.

### PsrA Is Able to Generate IR2- and IR3-Mediated Inversions *in vitro*

**Figure 10:**
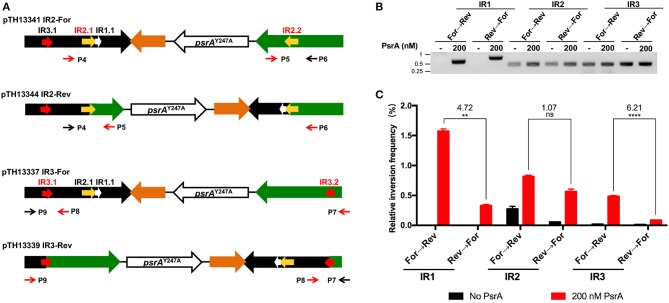
PsrA generates IR2 and IR3 inversions *in vitro*. **(A)** Relevant features of the plasmids used to analyse PsrA-mediated inversions between IR2.1 and IR2.2 and between IR3.1 and IR3.2. Primers P4/P5 and primers P5/P6 were used to detect IR2 inversions in pTH13341 and pTH13344, respectively. Primers P7/P8 and primers P8/P9 were used to detect IR3 inversions in pTH13337 and pTH13339, respectively. **(B)** IR1, IR2, and IR3 inversions detected by PCR assays (agarose gel). Plasmids used to analyse IR1 inversions are shown in Figure 8A. Plasmid DNA (1 nM) was incubated with PsrA (200 nM) at 37°C for 2 h in the presence of 7.5 mM MgCl_2_. The position of DNA molecular weight markers (in kb) is indicated on the left of the gel. **(C)** Relative frequency of IR1, IR2, and IR3 inversions determined by qPCR assays (bar graph). The result of inversion frequency was analyzed using two-tailed unpaired Student's *t* tests (means ± SEM). Statistical significance is defined by *P* values of <0.01 (**) or <0.0005 (****).

To investigate whether PsrA was able to generate IR2- and IR3-mediated inversions *in vitro*, we constructed four recombinant plasmids (Figure 10A). Plasmids pTH13341 (IR2-For) and pTH13344 (IR2-Rev) lack the IR1.2 and IR3.2 repeats. They were used to detect and quantify the occurrence of IR2 inversions by PCR and qPCR using primers P4/P5 (from “For” to “Rev,” pTH13341) or P5/P6 (from “Rev” to “For,” pTH13344). Plasmids pTH13337 (IR3-For) and pTH13339 (IR3-Rev) lack the IR1.2 and IR2.2 repeats. They allowed us to examine the occurrence of IR3 inversions using primers P7/P8 (from “For” to “Rev,” pTH13337) or P8/P9 (from “Rev” to “For,” pTH13339). In the inversion reactions shown in Figures 10B (PCR assays) and 10C (qPCR assays), we used 200 nM of PsrA since this concentration led to the highest frequency of IR1 inversions when 1 nM plasmid DNA, 125 mM NaCl and 7.5 mM MgCl_2_ were used (Figure S9). According to our previous *in vivo* studies (Li et al., 2019), PsrA-independent IR2- and IR3-mediated inversions may occur in *E. coli* with low frequency. Thus, the abovementioned plasmids isolated from *E. coli* were expected to have both configurations, “For” and “Rev,” as it was further confirmed when *in vitro* inversion reactions were carried out in the absence of PsrA (Figure 10B). Nevertheless, in the presence of PsrA (200 nM), we found a significant increase in the frequency of IR2- and IR3-mediated inversions (Figure 10C). Moreover, like in PsrA-mediated IR1 inversions (Figure 10C), the frequency of IR3 inversions from “For state” to “Rev sate” was higher than from “Rev state” to “For state,” whereas the frequency of IR2 inversions was similar in both directions (Figure 10C). These results demonstrated that PsrA catalyzes *in vitro* the formation of IR2- and IR3-mediated inversions in both directions.

## Discussion

Site-specific inversions in the *cod* locus of *S. pneumoniae* play an important role in the bacterial lifestyle by generation of epigenetic diversity and phenotypic phase variation in colony opacity that, in turn, leads to the adaptation of the pneumococci in colonization and virulence processes (Manso et al., 2014; Li et al., 2016; De Ste Croix et al., 2017). The gene encoding the PsrA Tyr-recombinase is included within the *cod* locus. *in vivo* studies showed that PsrA plays an essential role in promoting *hsdS* inversions between a pair of IR1 or IR1-like inverted repeats, with the exception of low-frequency sequence- and RecA-independent “spontaneous” inversions (Li et al., 2019). In this work, we have purified an untagged form of the PsrA protein and analyzed its DNA-binding capacities using different target DNAs. Furthermore, we have designed an approach to detect and quantify the DNA inversions generated by PsrA *in vitro*. This assay has allowed us to define the optimal conditions required for PsrA-mediated IR1 inversions.

In DNA-binding proteins, the location of their target sites on the genome is often preceded by an extensive search process, which results in transitory non-specific interactions between protein and DNA (Halford and Marko, 2004; Marcovitz and Levy, 2013). In the facilitated diffusion model (Berg et al., 1981; von Hippel and Berg, 1989), the protein first collides with the DNA at a random site (non-specific interactions) and then either locates its target site (specific interactions) or dissociates from the DNA. According to this model, sliding, and hopping events performed by the protein along the DNA regions surrounding the random site contribute to the search of the specific binding site. Our results suggest that PsrA might find its preferred target sites by a mechanism that involves initial binding at random DNA sites. First, by gel retardation assays, we found that PsrA binds to both linear dsDNAs and supercoiled DNAs containing or not inverted repeats. This behavior differs from other well-known Tyr-recombinases, such as Cre (Andrews et al., 1986; de Vargas et al., 1988; McCusker et al., 2007). However, further DNase I footprinting experiments performed with linear dsDNAs (265-bp) showed that PsrA binds to regions that contain either the IR1.1 or the IR1.2 repeat. On binding to these regions, PsrA generates higher-order complexes in which the DNA is not accessible to DNase I cleavage. Such nucleoprotein complexes do not enter the native PAA or agarose gels used in our EMSA experiments. Thus, to catalyze IR1-mediated inversions, PsrA could recognize the IR1 sequences and/or particular IR1-mediated structures. This conclusion is in agreement with our previous *in vivo* studies, which showed that PsrA-mediated IR1 inversions require both the 15-bp inverted repeats (IR1.1 and IR1.2) and the upstream adjacent sequences of both repeats (Li et al., 2019). Some reports support that site-specific recombinases should interact directly with inversely repeated sequences, and the position of the nucleophilic attack should be within the repeated sequence (Tong et al., 2014).

We have set up an *in vitro* procedure to detect PsrA-mediated DNA inversions. The procedure involves the use of an untagged form of PsrA and supercoiled plasmid DNA as target DNA. Using this *in vitro* assay, we have demonstrated that PsrA is able to catalyze three independent DNA inversion events mediated by IR1, IR2, and IR3 repeats, respectively. These findings support that PsrA locates specific binding sites on supercoiled DNAs and thus promote DNA inversions. The optimal conditions required for IR1-mediated inversions were found to be 37°C, pH 7.6, 125 mM NaCl, and 7.5 mM MgCl_2_. Interestingly, the frequency of PsrA-mediated IR1 inversions increased greatly in the presence of Mg^2+^. This cation could be partially replaced by Ba^2+^, Ca^2+^, and Fe^2+^ but not by Mn^2+^ or Zn^2+^. We hypothesize that Mg^2+^ could play a critical role in the catalytic reaction, although we cannot rule out the possibility of an effect on the PsrA oligomerization state and/or on the binding of PsrA to supercoiled plasmid DNAs. Usually, the active form of the Tyr-recombinases is a homodimer (Guo et al., 1997; Grindley et al., 2006). In the case of Cre, Mg^2+^ was shown to induce dimer formation and to stabilize Cre-Cre interactions in solution (Abremski and Hoess, 1984), even though the efficiency of the Cre-mediated catalytic reaction was Mg^2+^-independent (Ghosh et al., 2007). Additionally, structural studies have shown that mono- and multivalent ions influence the configuration of DNA molecules, and cations like Mg^2+^ can induce DNA condensation (Baumann et al., 1997; Bloomfield, 1997).

The three-dimensional structures of various recombinase-DNA complexes have been solved and no cations have been identified in the interface of the active site (Guo et al., 1997; Chen et al., 2000; Biswas et al., 2005). In addition to the catalytically active Tyr residue, five highly conserved amino acids participate in the catalytic center, namely one Lys, two Arg, and two His residues (or one His and one Trp in Cre and Flp). These residues generate a positively charged pocket that accommodates the negatively charged scissile phosphate group (Chen and Rice, 2003). Furthermore, several reports have shown that ‘classical’ Tyr-recombinases do not need a cation for catalysis (Ghosh et al., 2007; Fan, 2012). Thus, and to the best of our knowledge, the pneumococcal PsrA protein would represent the first instance of a Mg^2+^-dependent Tyr-recombinase. We speculate that Mg^2+^ could be placed either at the active center or at another domain of the protein; alternatively, Mg^2+^ could favor a conformational change in the DNA target to increase the catalytic efficacy of PsrA. Finally, whether accessory host factor(s) participates in the DNA inversions mediated by PsrA *in vivo* and, if so, how would they work is still unknown to us. Previous reports have shown that the two scenarios (independence or dependence of host factors) do exist: Cre recombinase from phage P1 and Flp recombinase from the 2 micron plasmid of *S. cerevisiae* do not need accessory factors (Grindley et al., 2006), whereas λ integrase relies on host factors like IHF, Fis, and Xis to promote recombination and to determine the directionality of the process (Landy, 1989). This would be also a matter of future exploration.

## Materials and Methods

### Bacterial Strains and Chemical Reagents

All strains used are listed in Table S1. *Escherichia coli* strains were grown in Luria-Bertani (LB) broth, or on LB agar plates, at 37°C under aerobic conditions. To overproduce PsrA, *E. coli* cells were grown in TY (tryptone-yeast extract) medium. Kanamycin (30 μg/ml) was added when needed. All primers were synthesized by Sangon Biotech (Beijing, China) and are listed in Table S2. PrimeSTAR® HS DNA Polymerase (TaKaRa Bio Inc, Kyoto, Japan) was used for polymerase chain reaction (PCR). PCR products were purified with the QIAquick PCR purification kit (QIAGEN, Hilden, Germany). All restriction enzymes were purchased from New England Biolabs (NEB; Beijing, China). All chemical reagents used in this work were purchased from Sigma (Shanghai, China) unless otherwise mentioned. Sanger sequencing data were obtained from Ruibiotech Co (Beijing, China) or from Secugen CIB (Madrid, Spain).

### Construction of Recombinant Plasmids

To overproduce PsrA, gene *psrA* was cloned into the inducible expression vector pET24b (Novagen, Beijing, China) harbored by *E. coli* BL21 (DE3) strain (a gift of F. W. Studier). This strain has the λDE3 lysogen that carries the gene for T7 RNA polymerase under control of the *lacUV5* promoter (Studier and Moffatt, 1986), which is inducible by isopropyl β-D-1-thiogalactopyranoside (IPTG). Plasmid pET24b carries the ∅10 promoter that is recognized by the T7 RNA polymerase. Gene *psrA* was amplified with primers pr15159 and pr15160 from ST606, digested with *Nde*I and *Xho*I, and inserted into the multiple cloning site (MCS) of pET24b to generate pTH12647 (pET24b::*psrA*).

Recombinant plasmids used for *in vitro* DNA inversion assays were constructed as described in Table S3. A pair of plasmids containing either the “For” or the “Rev” state were constructed for each pair of inverted repeats (IR1, IR2, and IR3) in a blunt-end cloning vector pEASY-blunt zero (TransGen Biotech, Beijing, China), which was then introduced into *E. coli* DH5α competent cells by heat shock as described before (Li et al., 2019). In each recombinant plasmid, only one pair of IRs was preserved. For instance, IR2.2 and IR3.2 were removed from the inserted S region of the *cod* locus in the pneumococcal strain TH6552 (*psrA*^Y247A^) (Li et al., 2019) to obtain TH8563 (*psrA*^Y247A^ΔIR2.2IR3.2) via JC1-insertion and counter selection. Briefly, upstream and downstream fragments were amplified with pr9803/pr11594 and pr10106/pr11595, digested with *Xba*I and *Xho*I, respectively, and then ligated with *Xba*I/*Xho*I-digested JC1 (modified Janus cassette, amplified with pr9840/pr1098) (Li et al., 2016); this generated element was introduced into *S. pneumoniae* ST606 by natural transformation to obtain TH8551 (*psrA*^Y247A^ΔIR2.2IR3.2::JC1) as previously described (Li et al., 2019); next, the two amplicons were both digested with *Xba*I, then ligated together, and transformed into TH8551 to obtain TH8563, whose chromosomal DNA was used as the template to construct pTH13166 (IR1-For) and pTH13170 (IR1-Rev), in which the orientations of the S region between IR1.1 and IR1.2 were opposite, represented as S1 and S2 configurations. Similarly, pTH13341 (IR2-For) and pTH13344 (IR2-Rev) were used for testing IR2 inversions, with IR1.2 and IR3.2 deleted, and S configurations were S1 and S3; pTH13337 (IR3-For) and pTH13339 (IR3-Rev), used for IR3 inversions, have S1 and S4 configurations, respectively, with IR1.2 and IR2.2 deleted. Pneumococcal chromosomal DNA was isolated by the DNeasy Blood & Tissue Kit (Qiagen, Hilden, Germany) as previously described (Feng et al., 2014). Plasmid DNA was prepared with HiPure Plasmid Plus Midi Kit (Magen, Beijing, China) according to manufacturer's instructions. All insertion elements were verified by Sanger sequencing.

### Overproduction and Purification of PsrA

To overproduce PsrA, *E. coli* BL21 (DE3) cells harboring plasmid pTH12647 were grown in TY medium supplemented with kanamycin (30 μg/ml) at 37°C and with aeration to mid-log phase [optical density at 600 nm (OD_600_) of 0.4–0.5]. Expression of the *psrA* gene was induced with IPTG (0.25 mM). After 25 min, bacterial cells were incubated with rifampicin (0.2 mg/ml) for 1 h. Rifampicin inhibits *E. coli* RNA polymerase but not T7 RNA polymerase. Cells were harvested by centrifugation, washed twice with ice-cold buffer S (10 mM Tris-HCl, pH 7.6, 1 mM DTT, 5% glycerol) containing 300 mM NaCl, and stored at −80°C. To purify PsrA, we designed a procedure based on previous studies (Solano-Collado et al., 2013; Ruiz-Cruz et al., 2018). Essentially, bacterial cells were concentrated (40x) in buffer S containing 300 mM NaCl and a protease inhibitor cocktail (Roche, Shanghai, China). Cells were disrupted using a French Pressure Cell, and the whole-cell extract was centrifuged to sediment cell debris. Nucleic acids were precipitated with 0.2% polyethyleneimine (PEI) (on ice for 30 min). After centrifugation, proteins recovered in the supernatant were precipitated with 70% saturated ammonium sulfate (on ice for 30 min). Precipitated proteins were collected by centrifugation, dissolved in buffer S containing 300 mM NaCl, and dialyzed against the same buffer at 4°C. Proteins were then loaded onto a HiTrap^TM^ Heparin HP column (GE Healthcare Bio-Sciences Corp., NJ, USA), and proteins with an affinity for heparin were eluted using a linear gradient of NaCl (300–800 mM). Fractions containing PsrA were identified by SDS-polyacrylamide (12%) gel electrophoresis (SDS-PAGE), pooled and dialyzed against buffer S containing 300 mM NaCl. PsrA preparations were then concentrated with Microsep^TM^ Advance Centrifugal Device (3K MWCO) (Pall Life Sciences, NY, USA). Protein concentration was determined with the BCA assay kit (Solarbio, Beijing) and using a NanoDrop ND-2000 Spectrophotometer (Thermo Scientific, USA).

### Radiolabeling of DNA Fragments

Primers pr14684 and pr14687 were radioactively labeled at the 5′ end using T4 polynucleotide kinase (T4 PNK, NEB) and [γ-^32^P]-ATP (PerkinElmer Inc, USA). Reactions (25 μl) contained 25 pmol primer, 10 units T4 PNK, 50 pmol [γ-^32^P]-ATP (3,000 Ci/mmol, 10 mCi/ml), and kinase buffer (1x), which was provided by the supplier. After 30 min at 37°C, 10 units T4 PNK was added and reactions were incubated again at 37°C for 30 min. T4 PNK was inactivated (65°C, 20 min) and non-incorporated [γ-^32^P]-ATP was removed by passing through an Illustra MicroSpin^TM^ G-25 column (GE Healthcare). 5′-radiolabeled pr14684 and non-labeled pr14685 were used to generate the radiolabeled IR1.1 dsDNA fragment by PCR amplification; 5′-radiolabeled pr14687 and non-labeled pr14686 were used to generate the radiolabeled IR1.2 dsDNA fragment. The labeled pr14684 and pr14687 primers were also used to perform sequencing reactions with a 1067-bp IR1.1 fragment (pr14682/pr14685) and a 1009-bp IR1.2 fragment (pr14686/pr15159), respectively, by USB® Sequenase Version 2.0 DNA Sequencing Kit (Affymetrix Inc, Ohio, USA).

### Electrophoretic Mobility Shift Assays (EMSA)

The DNA fragments used for EMSA were amplified with the following primer pairs: IR3.1-pr14682/pr14683, IR1.1-pr14684/pr14685, IR1.2-pr14686/pr14687, and *hsdM*-pr14691/pr14692 from ST606. Short-length dsDNA fragments of 26-bp (pr15163/pr15164) were obtained by oligonucleotide annealing (Solano-Collado et al., 2013). EMSA reactions were basically performed in the following conditions: 30 mM Tris-HCl, pH 7.6, 1 mM DTT, 0.25 mM EDTA, 125 mM NaCl, 0.5 mg/ml bovine serum albumin (BSA), 10 mM MgCl_2_, 1 mM CaCl_2_, 1.25% glycerol, 10 nM non-labeled DNA or 2 nM ^32^P-labeled IR1.2 DNA, and various concentrations of PsrA. Reactions were incubated at room temperature for 20 min, followed by mixing with 10 × BXGE loading buffer (0.25% bromophenol blue, 0.25% xylene cyanol, 60% glycerol, and 10 mM EDTA). Reactions with non-labeled DNA were analyzed by electrophoresis on native PAA (6%) gels in TBE (Tris-borate-EDTA, pH 8.3) buffer or on agarose (0.6% to 2%) gels in TAE (Tris-acetate-EDTA, pH 8.3) buffer. Non-labeled DNA was visualized with Gel-Red (Biotium) staining under a ChemiDoc™ XRS+ System (Bio-Rad) and quantified with ImageLab software (Bio-Rad). Reactions with radiolabeled DNA were also analyzed by electrophoresis on native PAA (6%) gels, but DNA was visualized using a Fujifilm Image Analyzer FLA-3000 and quantified using the Quantity One software (Bio-Rad) (Solano-Collado et al., 2016). Competitive EMSA reactions were performed with 2 nM ^32^P-labeled IR1.2 DNA and increasing concentrations of non-labeled calf thymus DNA (Thermo Fisher Scientific, USA). Dissociation of PsrA-IR1.2 complexes was achieved by adding non-labeled IR1.2 or a 266-bp control DNA (amplified with pr15161 and pr15162 from *E. faecalis* OG1RF (a gift from Ana Moreno-Blanco at CIB, CSIC, Spain), and incubating for another 5 min before analyzing by 6% PAGE.

### DNase I Footprinting Assays

PsrA-DNA binding reactions (8 μl) contained 30 mM Tris-HCl, pH 7.6, 1 mM DTT, 0.25 mM EDTA, 125 mM NaCl, 0.5 mg/ml BSA, 10 mM MgCl_2_, 1 mM CaCl_2_, 1.25% glycerol, 4 nM radiolabeled IR1.2 DNA or 6 nM radiolabeled IR1.1 DNA, and different concentrations of PsrA. Reactions were incubated at room temperature for 20 min. Then, protein-DNA complexes were treated with DNase I (0.01 units) for 5 min at the same temperature. DNase I digestion was stopped by adding 1 μl of 250 mM EDTA. Four microlitres of loading buffer (80% formamide, 1 mM EDTA, 10 mM NaOH, 0.1% bromophenol blue, and 0.1% xylene cyanol) was added to the reaction mixtures. Samples were heated at 95°C for 5 min, and immediately chilled on ice before loading onto 8 M urea-6% polyacrylamide gels. After running, gels were dried and exposed to a Phosphorimager screen (Fujifilm, Japan). The radioactive intensity was visualized by a Fujifilm Image Analyser FLA-3000 and was quantified using the Quantity One software (Bio-Rad) (Solano-Collado et al., 2013; Ruiz-Cruz et al., 2018).

### *In vitro* DNA Inversion Assays

The basic components of the *in vitro* DNA inversion reactions are listed as followed: 25 mM Tris-HCl, pH 7.6, 1 mM DTT, 1 mM EDTA, 125 mM NaCl, 5 mM MgCl_2_, 1 nM DNA, and 125 nM PsrA, referring to previous studies on several classical tyrosine recombinases (Parsons et al., 1990; Ringrose et al., 1998; Sandal et al., 2001; Tong et al., 2014). Reactions (20 μl) were started by adding PsrA protein, and performed at 37°C for 1 h. 80 μl ddH_2_O was then added to stop the reaction and reactions were 20-fold diluted in ddH_2_O for subsequent analysis and quantification by PCR and quantitative real-time PCR (q-PCR), respectively. Primer pairs used for testing DNA inversions of different substrates were as followed: P1/P2-pTH13366; P1/P3-pTH13370; P4/P5-pTH13341; P5/P6-pTH13344; P7/P8-pTH13337, and P8/P9-pTH13339. PCR was performed with the primer pairs mentioned above and analyzed by 1% agarose gel electrophoresis. qPCR was used to calculate the relative inversion frequency by normalizing each Δ*C*_T_ value (*C*_T_ of inversion reaction minus *C*_T_ of reference gene *era*, amplified with primers pr7932 and pr10129) with Δ*C*_T_ of the reaction at 37°C for 1 h containing basic components.

To calculate absolute inversion frequency of IR1, IR2, and IR3-mediated inversions *in vitro*, two pairs of primers were used to amplify “For” and “Rev” states, respectively. pTH13366: For-P1/P3, Rev-P1/P2; pTH13370: For-P1/P2, Rev-P1/P3; pTH13341: For-P5/P6, Rev-P4/P5; pTH13344: For-P4/P5, Rev-P5/P6; pTH13337: For-P8/P9, Rev-P7/P8; pTH13339: For-P7/P8, Rev-P8/P9. Like in qPCR to calculate relative inversion frequency, individual *C*_T_ values of For and Rev state were first normalized by subtracting the *C*_T_ of the reference gene *era* to obtain Δ*C*_T_. Δ*C*_T_ values of For and Rev states were normalized again with the For state to obtain Δ*ΔC*_T−For_ and Δ*ΔC*_T−Rev_. The final amplification level of For and Rev states was 2^−ΔΔ*C*T−*For*^ and 2^−ΔΔ*C*T−*Rev*^, and the absolute inversion frequency was the ratio of 2^−ΔΔ*C*T−*Rev*^ in the total value of 2^−ΔΔ*C*T−*For*^ and 2^−ΔΔ*C*T−*Rev*^.

### Statistical Analysis

Statistical analysis was performed using the GraphPad Prism 7.0 software (San Diego, CA). The qPCR data of *in vitro* inversion frequency were also analyzed using two-tailed unpaired Student's *t* tests (means ± standard error of the mean). Statistical significance is defined by *P* values of < 0.05 (^*^), < 0.01 (^**^), < 0.001(^***^), or < 0.0005 (^****^).

## Data Availability Statement

All datasets generated for this study are included in the article/Supplementary Material.

## Author Contributions

This study was designed by ME, AB, and J-RZ. Laboratory work was performed by JL, JW, and SR-C. The results were analyzed and discussed between all authors. The first draft of the manuscript was elaborated by JL, JW, ME, and AB. Several copies of the different versions of the manuscript were circulated among all the authors. The final version of the manuscript was done by AB and J-RZ. All authors read and approved the final manuscript.

### Conflict of Interest

The authors declare that the research was conducted in the absence of any commercial or financial relationships that could be construed as a potential conflict of interest.
