# Supplementary figures and images for: In vitro DNA Inversions Mediated by the PsrA Site-Specific Tyrosine Recombinase of Streptococcus pneumoniae
Source: Front Mol Biosci. 2020 Mar 19;7:43. doi: 10.3389/fmolb.2020.00043 (PMC7096588; doi:10.3389/fmolb.2020.00043)

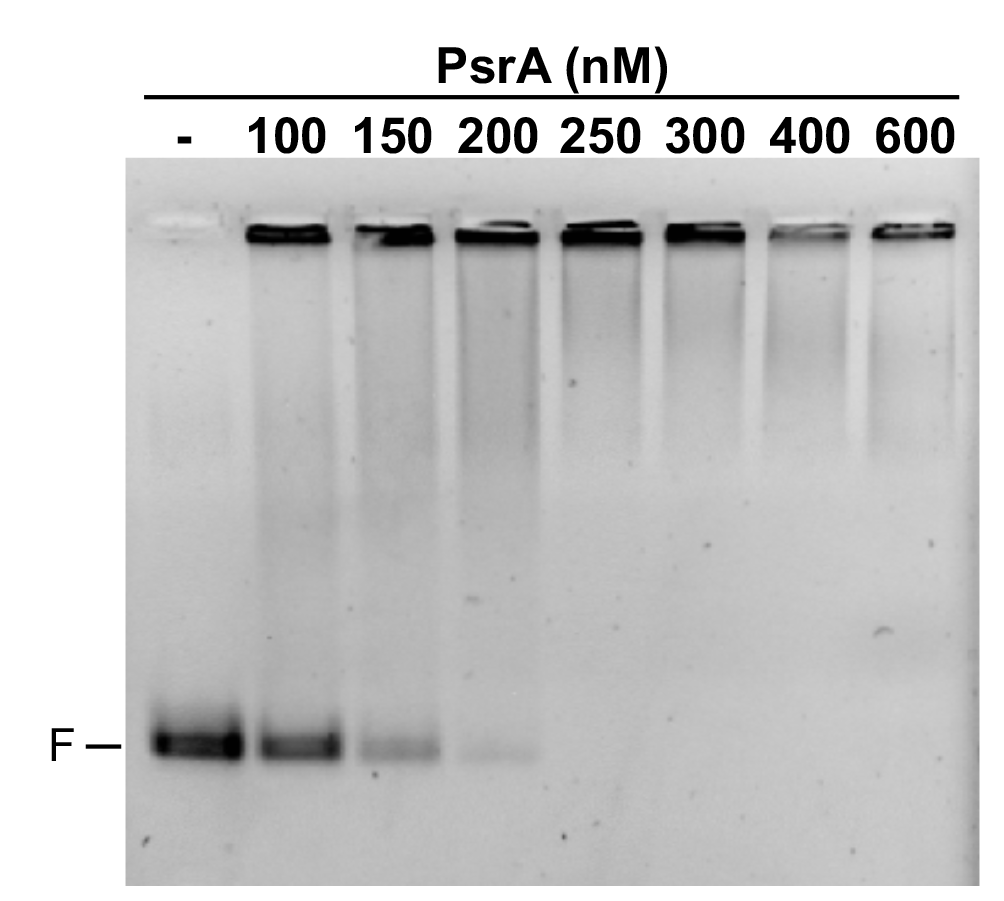

Supplement: Supplementary file 1 [file Image_1.TIF]

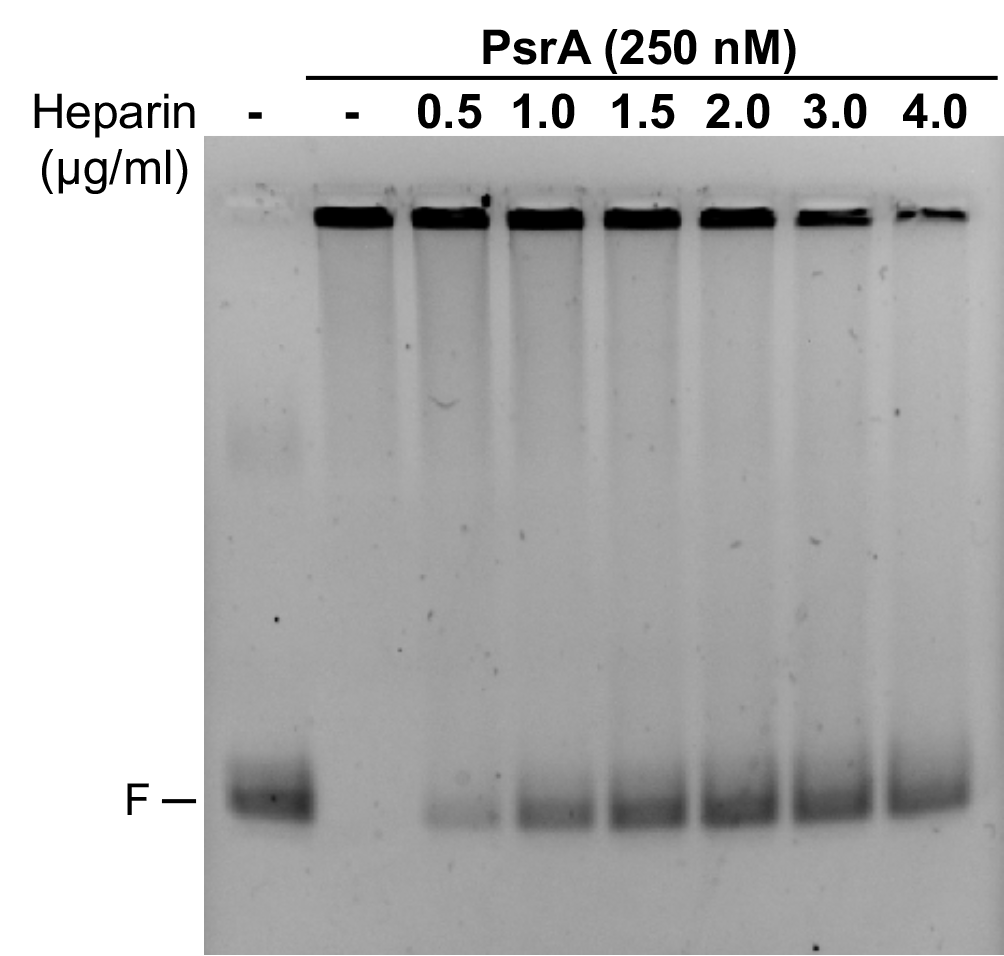

Supplement: Supplementary file 2 [file Image_2.TIF]

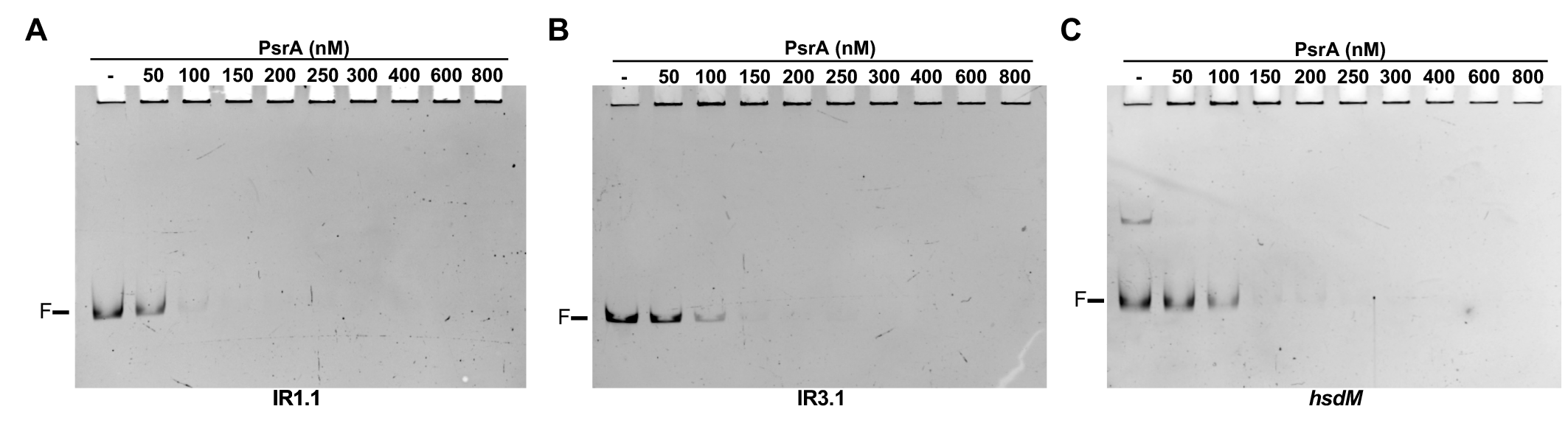

Supplement: Supplementary file 3 [file Image_3.TIF]

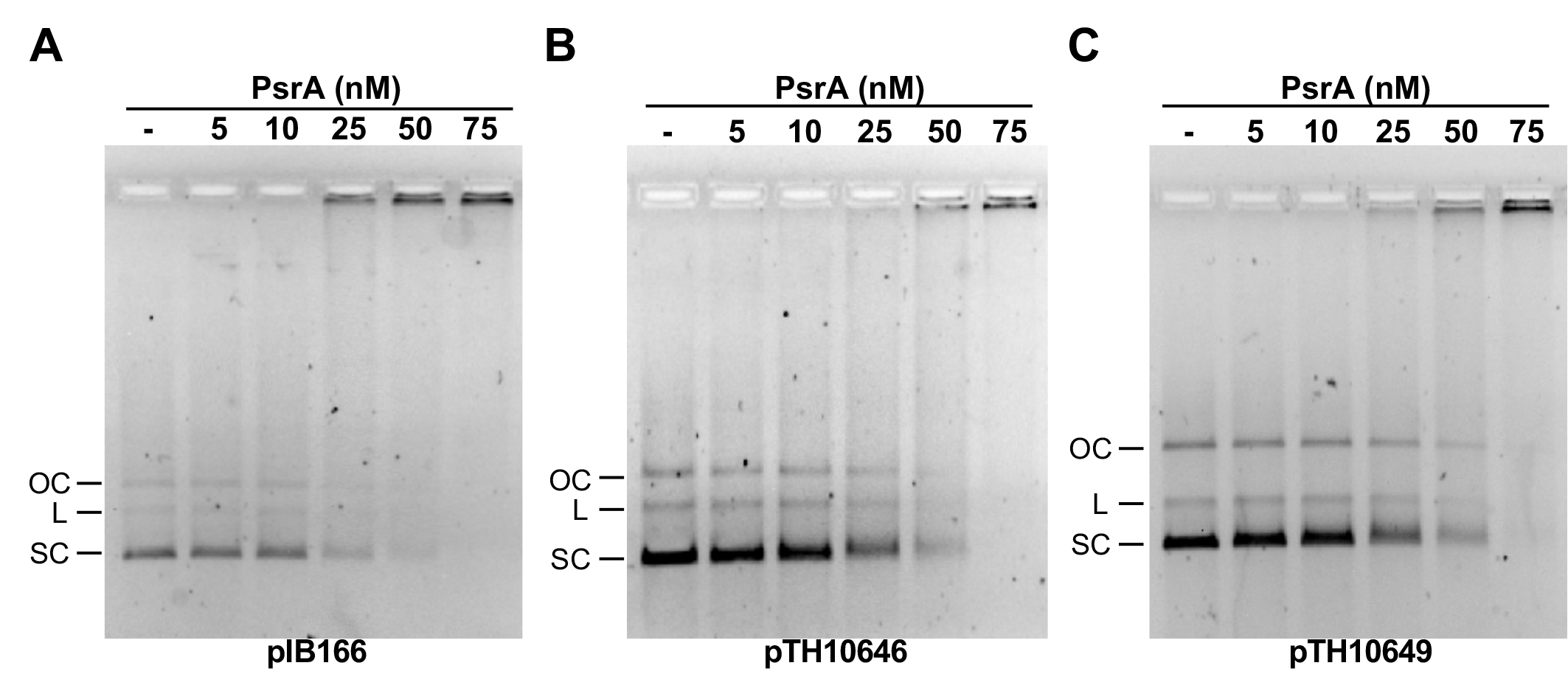

Supplement: Supplementary file 4 [file Image_4.TIF]

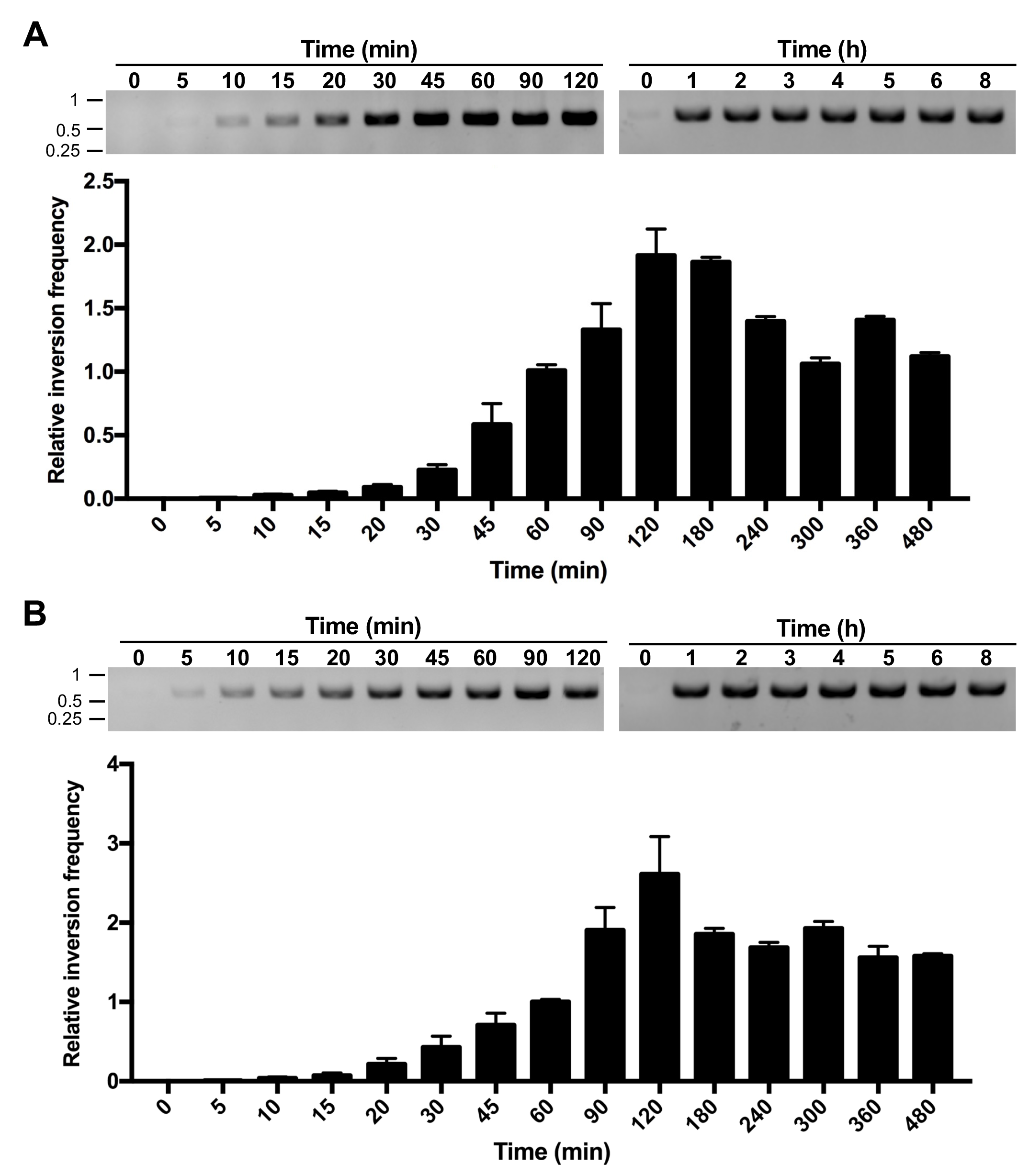

Supplement: Supplementary file 5 [file Image_5.TIF]

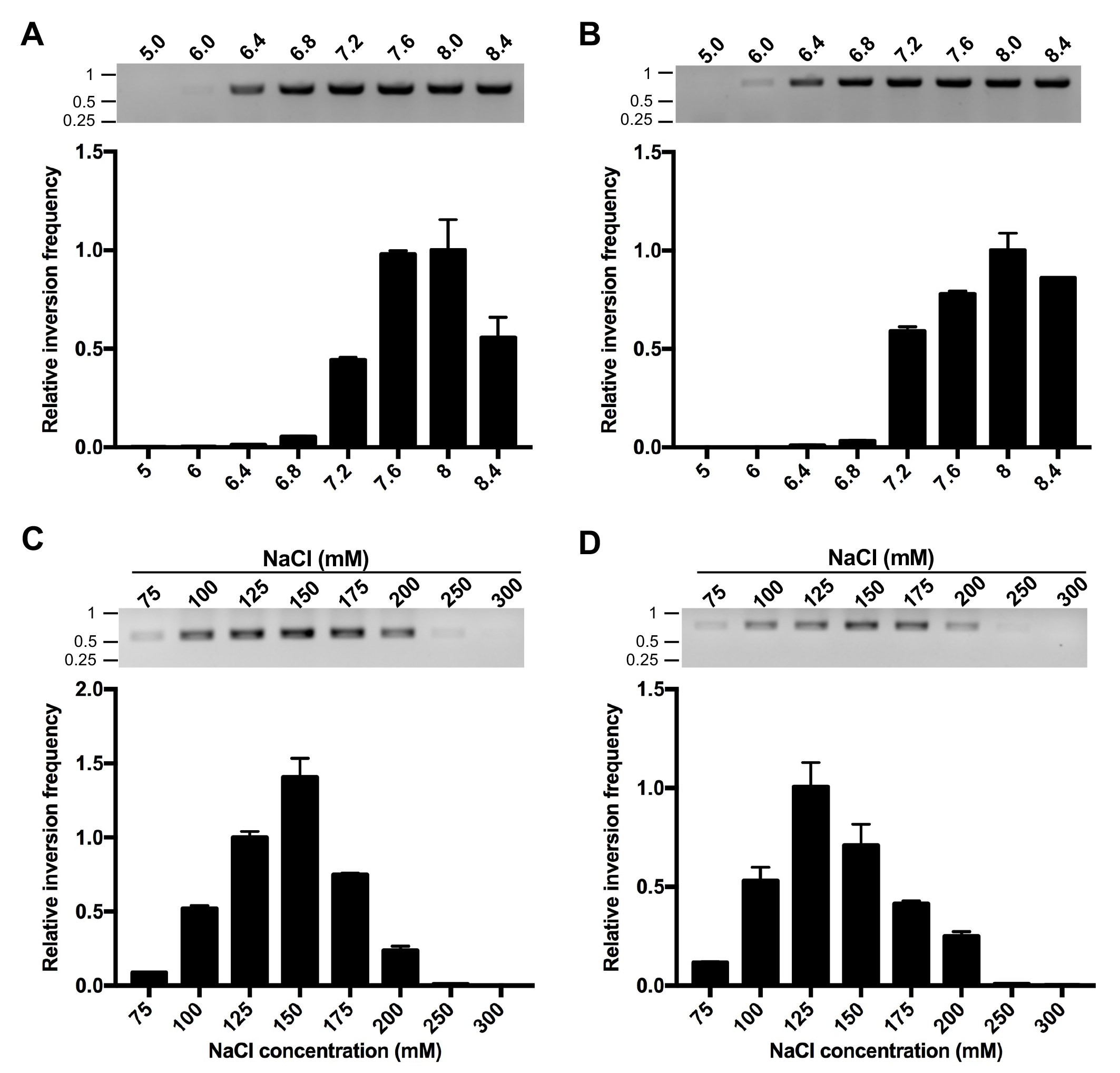

Supplement: Supplementary file 6 [file Image_6.TIF]

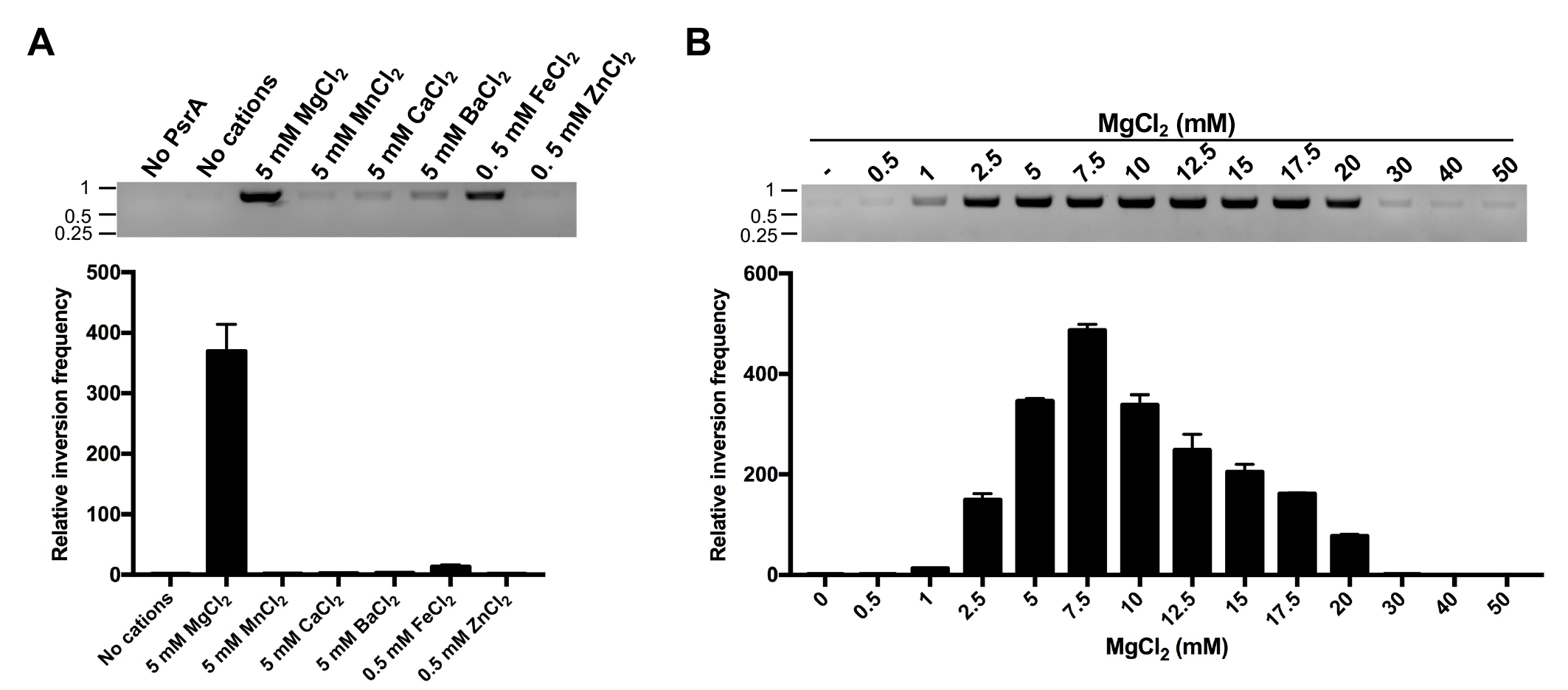

Supplement: Supplementary file 7 [file Image_7.TIF]

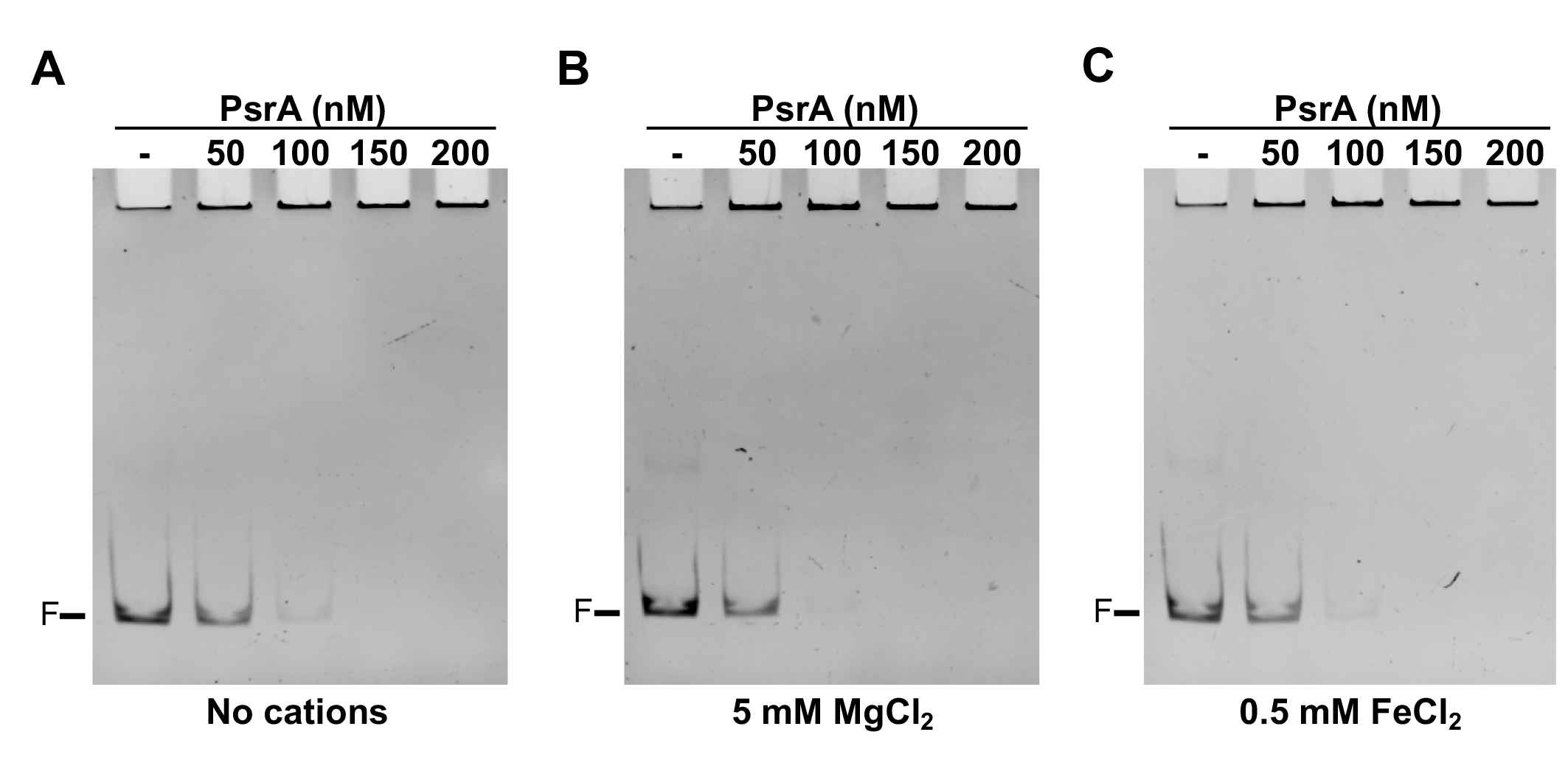

Supplement: Supplementary file 8 [file Image_8.TIF]

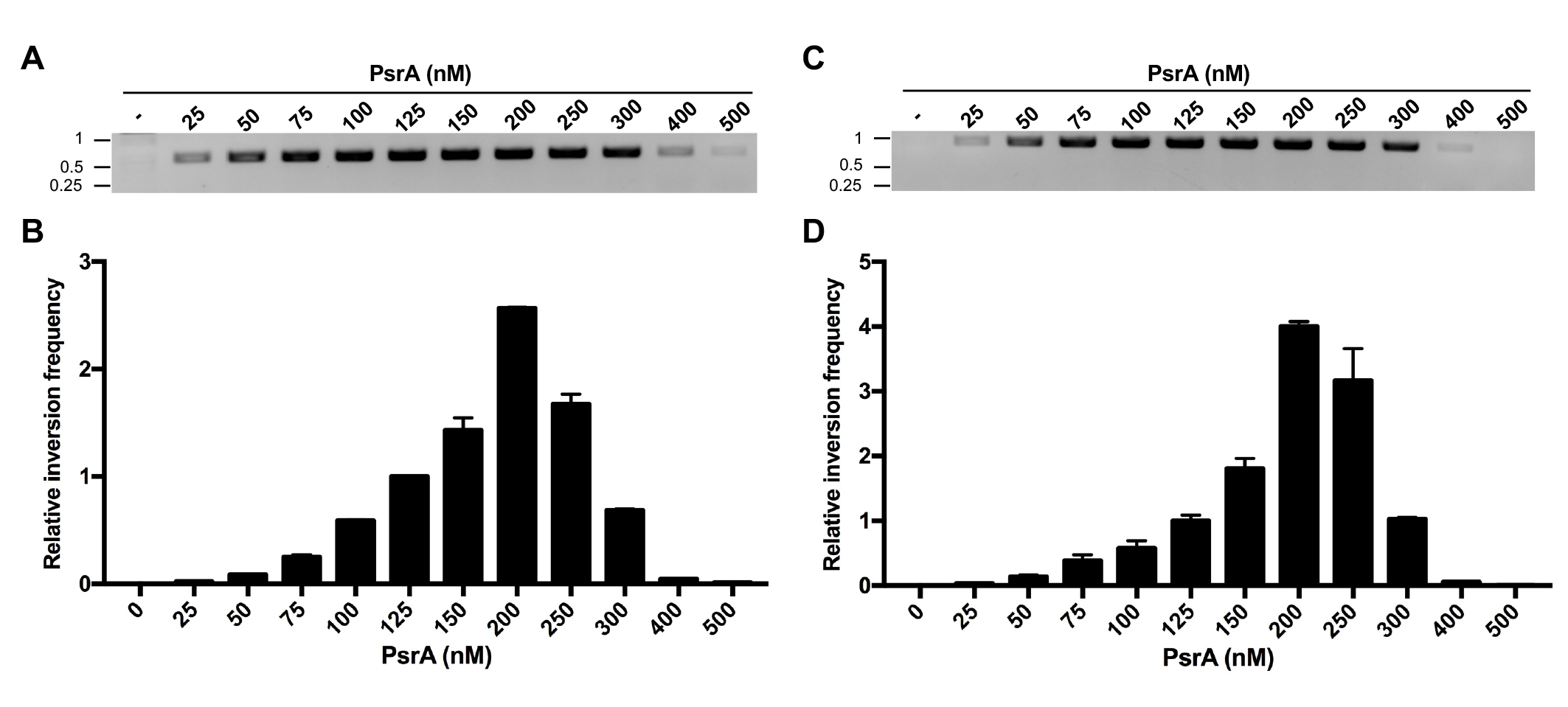

Supplement: Supplementary file 9 [file Image_9.TIF]
