# Supplementary material for: In vitro DNA Inversions Mediated by the PsrA Site-Specific Tyrosine Recombinase of Streptococcus pneumoniae
Source: Front Mol Biosci. 2020 Mar 19;7:43. doi: 10.3389/fmolb.2020.00043 (PMC7096588; doi:10.3389/fmolb.2020.00043)
Supplement: Supplementary file 10 [file Table_1.DOCX]

**Table S1. Bacterial strains used in this study**

| **Strain** | **Description** | **Antibiotic resistance** | **Reference or source** | |
| --- | --- | --- | --- | --- |
| ***E. coli*** | | | |  |
| DH5α | F^–^ *endA1* *glnV44* *thi-1* *recA1* *relA1* *gyrA96* *deoR* *nupG* *purB20* φ80d*lacZ*ΔM15 Δ(*lacZYA-argF*)U169, *hsdR17*(*r_K_*^–^*m_K_*^+^), λ^–^ | / | (Hanahan, 1983) |  |
| BL21 (DE3) | str. B F^–^ *ompT* *gal* *dcm* *lon* *hsdS_B_*(*r_B_*^–^*m_B_*^–^)  λ(DE3 [*lacI* *lacUV5*-*T7p07* *ind1* *sam7* *nin5]*) [*malB*^+^]_K-12_(λ^S^) | / | (Studier and Moffatt, 1986) |  |
| TH12647 | BL21 (DE3); carrying pET24b::*psrA* | Kan^R^ | This study |  |
| TH13166 | DH5α; carrying pEASY blunt zero::Spn556IIS1-*psrA*^Y247A^-∆IR2.2∆IR3.2 | Amp^R^ Kan^R^ | This study |  |
| TH13170 | DH5α; carrying pEASY blunt zero::Spn556IIS2-*psrA*^Y247A^-∆IR2.2∆IR3.2 | Amp^R^ Kan^R^ | This study |  |
| TH13337 | DH5α; carrying pEASY blunt zero::Spn556IIS1-*psrA*^Y247A^-∆IR1.2∆IR2.2 | Amp^R^ Kan^R^ | This study |  |
| TH13339 | DH5α; carrying pEASY blunt zero::Spn556IIS4-*psrA*^Y247A^-∆IR1.2∆IR2.2 | Amp^R^ Kan^R^ | This study |  |
| TH13341 | DH5α; carrying pEASY blunt zero::Spn556IIS3-*psrA*^Y247A^-∆IR1.2∆IR3.2 | Amp^R^ Kan^R^ | This study |  |
| TH13344 | DH5α; carrying pEASY blunt zero::Spn556IIS1-*psrA*^Y247A^-∆IR1.2∆IR3.2 | Amp^R^ Kan^R^ | This study |  |
| ***S. pneumoniae*** | | | |  |
| ST556 | serotype 19F, a multidrug-resistant isolate from a patient with otitis media | / | (Li et al., 2012) |  |
| ST606 | ST556 derivative; *rpsL1* | Sm^R^ | (Li et al., 2016) |  |
| TH6552 | *psrA*^Y247A^ | Sm^R^ | (Li et al., 2019) |  |
| TH8551 | *psrA*^Y247A^∆IR2.2IR3.2::JC1 | Sm^R^ | This study |  |
| TH8563 | *psrA*^Y247A^∆IR2.2IR3.2 | Sm^R^ | This study |  |
| TH7377 | *psrA*^Y247A^∆IR1.2 | Sm^R^ | (Li et al., 2019) |  |

**References:**

Hanahan, D. (1983). Studies on transformation of Escherichia coli with plasmids. *J Mol Biol* 166(4)**,** 557-580.

Li, G., Hu, F.Z., Yang, X., Cui, Y., Yang, J., Qu, F., et al. (2012). Complete genome sequence of Streptococcus pneumoniae strain ST556, a multidrug-resistant isolate from an otitis media patient. *J Bacteriol* 194(12)**,** 3294-3295. doi: 10.1128/JB.00363-12.

Li, J., Li, J.W., Feng, Z., Wang, J., An, H., Liu, Y., et al. (2016). Epigenetic Switch Driven by DNA Inversions Dictates Phase Variation in Streptococcus pneumoniae. *PLoS Pathog* 12(7)**,** e1005762. doi: 10.1371/journal.ppat.1005762.

Li, J.W., Li, J., Wang, J., Li, C., and Zhang, J.R. (2019). Molecular Mechanisms of hsdS Inversions in the cod Locus of Streptococcus pneumoniae. *J Bacteriol* 201(6)**,** pii: e00581-00518. doi: 10.1128/JB.00581-18.

Studier, F.W., and Moffatt, B.A. (1986). Use of bacteriophage T7 RNA polymerase to direct selective high-level expression of cloned genes. *J Mol Biol* 189(1)**,** 113-130.
