# Supplementary material for: In vitro DNA Inversions Mediated by the PsrA Site-Specific Tyrosine Recombinase of Streptococcus pneumoniae
Source: Front Mol Biosci. 2020 Mar 19;7:43. doi: 10.3389/fmolb.2020.00043 (PMC7096588; doi:10.3389/fmolb.2020.00043)
Supplement: Supplementary file 11 [file Table_2.DOCX]

**Table S2. Primers used in this study**

| **Primers** | **Sequence (5'-3')** |
| --- | --- |
| P1 (pr12011) | GTTAATTGGTAAAAAATAACAGGTGGTCAAACTG |
| P2 (pr12013) | CGGAATTAATAGCTCGCTTAGTGTAG |
| P3 (pr12012) | CTACACTAAGCGAGCTATTAATTCCG |
| P4 (pr14929) | AACTTTCTGGTATTTCACAAGGTACTTCC |
| P5 (pr14930) | AGTGGAGCTGTTGTGAAAAACTTGAATAG |
| P6 (pr14931) | ACTGGAACAAGTTATCCTGCAATCAATG |
| P7 (pr14926) | TGCACCATCAACATTCAAAATATAAGG |
| P8 (pr14927) | TATCAGTGAAGGAAAAATCAAACGAGAT |
| P9 (pr14928) | CTATTTCAACCCCATTATCTATAGGCG |
| pr1098 | GAGACTCGAGCCTTTCCTTATGCTTTTGGAC |
| pr7566 | ATGACACCAGAACAACTTAAAGCAAGTATTCT |
| pr7567 | ATTATCTACAATCTCATCAACAATAAACCTATTTCAA |
| pr7932 | GATTGCCATCATGAGTGACAAGG |
| pr9803 | TTAGGAATGTACAGTCTACGAATTTTACCACC |
| pr9840 | GAGATCTAGAGGATAATGCTGAAAACTCCTTGAAG |
| pr10106 | ACCCTTAAAATAGCGATTTCGGTCG |
| pr10129 | TGATCAGACGGGAAATATTGGAAACC |
| pr11594 | GAGATCTAGAGTACCTTGTGAAATACCAGAAAGTTGG |
| pr11595 | GAGACTCGAGTCTAGATTATGTGCTTCAAGTATATAAAAAGGCG |
| pr14682 | CAATGACGAACCTGCAAGTGAAT |
| pr14683 | AATCTTTGATTGGTCGTGGAGAG |
| pr14684 | CCAAATGATGAATCAGTCGAAGTTT |
| pr14685 | TATTACCACCACGTATAATTCTAACACCT |
| pr14686 | CCCAAAAAGTAGCTTCACTGCG |
| pr14687 | TAGAAACTACTCGAATTTATCTAAGGAAAAC |
| pr14691 | GGAGAATCAATGGCAGACCTTGC |
| pr14692 | TAATAATGTCAAATTTTTCATCATCCG |
| pr14885 | GGTCTCTTAATAAGGGCAATTCTGCAGATATCCA |
| pr14886 | GGTCTCTTCATAAGGGCAATTCCAGCACACTG |
| pr14887 | GGTCTCTATGACACCAGAACAACTTAAAGCAAG |
| pr14888 | GGTCTCTAGGAAGTACCTTGTGAAATACCAGAAAG |
| pr14889 | GGTCTCTTCCTGGATAGTGGGGGGAGAGCAAT |
| pr14890 | GGTCTCTATTATCTACAATCTCATCAACAATAAACCTATT |
| pr14891 | GGTCTCTTTATCCTCACCCACTCCCAAGTATCAG |
| pr14892 | GGTCTCTATAAAATCAATTTATTGGAATTTTGGG |
| pr14893 | GGTCTCTTTTTCTACATTGGTTGAAATTGTCAGAG |
| pr14894 | GGTCTCTAAAACCTCACCCACTCCCAAGTATCAG |
| pr14895 | GGTCTCTATAAATAAAATCAATTTATTGGAATTTTGGG |
| pr14896 | GGTCTCTTTATGTGCTTCAAGTATATAAAAAGGCG |
| pr14897 | GGTCTCTTCCTCATAATAAGAGTTATCATCTCCTTGGGAA |
| pr14898 | GGTCTCTAGGAAGTACCTTGTGAAATACCAGAAAG |
| pr14899 | GGTCTCTGGAATATACCTATGAATTGGGTTGTTATA |
| pr14900 | GGTCTCTTTCCCATAATAAGAGTTATCATCTCCTTGGGAA |
| pr15011 | GGTCTCTCAAAGACGAAACAACCTCTTATCC |
| pr15012 | GGTCTCTTATACCTATGAATTGGGTTGTTATAAAAAT |
| pr15013 | GGTCTCTTTTGTTCCCATAATAAGAGTTATCATCTCCTTG |
| pr15014 | GGTCTCTTATATTCCCATAATAAGAGCCATAATCACA |
| pr15159 | CATAAGATAGGAGTTTTCATATGAAAGATTTTG |
| pr15160 | GTAGTCAGTTTGACCTCGAGTTATTTTTTACC |
| pr15161 | GCAAACTGTTTCCAGTGATAGT |
| pr15162 | CCACAAACAACGTCGCTCCATCA |
| pr15163 | TTCTTTGTGGTATAATTGCAAGAGGT |
| pr15164 | ACCTCTTGCAATTATACCACAAAGAA |
