# Supplementary material for: In vitro DNA Inversions Mediated by the PsrA Site-Specific Tyrosine Recombinase of Streptococcus pneumoniae
Source: Front Mol Biosci. 2020 Mar 19;7:43. doi: 10.3389/fmolb.2020.00043 (PMC7096588; doi:10.3389/fmolb.2020.00043)
Supplement: Supplementary file 12 [file Table_3.DOCX]

**Table S3. Construction of recombinant plasmids**

| **Plasmid** | **Insertion elements** | **Parent vector** | **Target sequence** | **Primers** | **Templete DNA** | **Restrict sites** |
| --- | --- | --- | --- | --- | --- | --- |
| pTH12647 | *psrA* | pET24b | *psrA* | pr15159/pr15160 | ST606 | NdeI/XhoI |
| pTH13166 | Spn556IIS1-*psrA*^Y247A^-∆IR2.2∆IR3.2 | pEASY-blunt zero | Spn556IIS1-*psrA*^Y247A^-∆IR2.2∆IR3.2 | pr7566/pr7567 | TH8563 | Blunt cloning |
| pTH13170 | Spn556IIS2-*psrA*^Y247A^-∆IR2.2∆IR3.2 | pEASY-blunt zero | IR1.1-pEASY blunt zero-IR1.2 | pr15013/pr15014 | pTH13166 | BsaI |
|  |  |  | S between IR1.1 and IR1.2 | pr15011/pr15012 | TH8563 |  |
| pTH13337 | Spn556IIS1-*psrA*^Y247A^-∆IR1.2∆IR2.2 | pEASY-blunt zero | pEASY-blunt zero | pr14885/pr14886 | pTH13166 | BsaI |
|  |  |  | *hsdS_A_*-*hsdS_B_*∆IR1.2-*psrA*-*hsdS_C_* before IR2.2 | pr14887/pr14888 | TH7377 |  |
|  |  |  | *hsdS_C_* after IR2.2 | pr14889/pr14890 | TH7377 |  |
| pTH13339 | Spn556IIS4-*psrA*^Y247A^-∆IR1.2∆IR2.2 | pEASY-blunt zero | pEASY-blunt zero | pr14885/pr14886 | pTH13166 | BsaI |
|  |  |  | *hsdS_A_*-IR3.1 | pr14887/pr14891 | TH7377 |  |
|  |  |  | *hsdS_C_* between IR2.2 and IR3.2 | pr14892/pr14889 | TH7377 |  |
|  |  |  | S region between IR2.2 and IR3.1 | pr14888/pr14893 | TH7377 |  |
|  |  |  | IR3.2-*hsdS_C_* | pr14894/pr14890 | TH7377 |  |
| pTH13341 | Spn556IIS3-*psrA*^Y247A^-∆IR1.2∆IR3.2 | pEASY-blunt zero | pEASY-blunt zero | pr14885/pr14886 | pTH13166 | BsaI |
|  |  |  | *hsdS_A_*-*hsdS_B_*∆IR1.2-*psrA*-*hsdS_C_* before IR3.2 | pr14887/pr14895 | TH7377 |  |
|  |  |  | *hsdS_C_* after IR3.2 | pr14895/pr14890 | TH7377 |  |
| pTH13344 | Spn556IIS1-*psrA*^Y247A^-∆IR1.2∆IR3.2 | pEASY-blunt zero | pEASY-blunt zero | pr14885/pr14886 | pTH13166 | BsaI |
|  |  |  | *hsdS_A_*-IR2.1 | pr14887/pr14897 | TH7377 |  |
|  |  |  | S region between IR2.1 and IR2.2 | pr14898/pr14899 | TH7377 |  |
|  |  |  | IR2.2-*hsdS_C_* before IR3.2 | pr14900/pr14895 | TH7377 |  |
|  |  |  | *hsdS_C_* after IR3.2 | pr14896/pr14890 | TH7377 |  |
