# Supplementary material for: In vitro DNA Inversions Mediated by the PsrA Site-Specific Tyrosine Recombinase of Streptococcus pneumoniae
Source: Front Mol Biosci. 2020 Mar 19;7:43. doi: 10.3389/fmolb.2020.00043 (PMC7096588; doi:10.3389/fmolb.2020.00043)
Supplement: Supplementary file 13 [file Data_Sheet_1.DOCX]

**Figure S1. EMSA of IR1.2-PsrA complexes.** Non-labelled IR1.2 DNA (10 nM) was incubated with the indicated concentrations of PsrA. Binding reactions were analysed by agarose (1%) gel electrophoresis. F: free DNA.

**Figure S2. Effect of heparin on the formation of IR1.2-PsrA complexes.** Non-labelled IR1.2 DNA (10 nM) was incubated with PsrA (250 nM). Then, heparin was added at the indicated concentrations. Binding reactions were loaded onto a TAE-agarose (0.6%) gel. F: free DNA.

**Figure S3. Binding reactions using different linear dsDNA fragments (IR1.1, IR3.1 and *hsdM*)**. Non-labelled DNA (10 nM) was incubated with the indicated concentrations of PsrA. Binding reactions were loaded onto a native PAA (6%) gel. F: free DNA.

**Figure S4. Binding reactions using different supercoiled plasmid DNAs.** The indicated plasmids (0.25 nM) were incubated with different concentrations of PsrA. **(A)** pIB166, empty plasmid. **(B)** pTH10646, IR1-spectinomycin reporter vector containing a pair of IR1 (IR1.1-*aad9*-IR1.2). **(C)** pTH10649, negative control vector of IR1-spectinomycin reporter containing only the left IR1 (IR1.1-*aad9*). Reaction mixtures were loaded onto TAE-agarose (0.6%) gels. SC: supercoiled, L: linear, OC: open circle.

**Figure S5. Relative frequency of PsrA-mediated IR1 inversions as a function of the incubation time.** Plasmids pTH13166 **(A)** and pTH13170 **(B)** were used (see Figure 8A). Plasmid DNA (1 nM) was incubated with PsrA (125 nM) at 37ºC for different times. Reaction mixtures contained NaCl (125 mM) and MgCl_2_ (5 mM). The occurrence of IR1 inversions was monitored by PCR assays (agarose gels) and by qPCR assays (bar graphs). The position of DNA molecular weight markers (in kb) is indicated on the left of the gels.

**Figure S6. Effect of pH and NaCl concentration on the frequency of PsrA-mediated IR1 inversions. (A)** and **(B)** Relative frequency of IR1 inversions (qPCR assays, bar graphs) in the presence of PsrA (125 nM) at different pH conditions using pTH13166 (1 nM) or pTH13170 (1 nM), respectively (see also Figure 8A). **(C)** and **(D**) Relative frequency of IR1 inversions (qPCR assays, bar graphs) in the presence of PsrA (125 nM) at different concentrations of NaCl using pTH13166 (1 nM) or pTH13170 (1 nM), respectively. Reaction mixtures contained MgCl_2_ (5 mM). Reactions were incubated at 37ºC for 1 h. IR1 inversions were also detected by PCR assays (agarose gels).

**Figure S7.** **Mg^2+^ enhances the frequency of PsrA-mediated IR1 inversions on pTH13170. (A)** Relative frequency of PsrA-mediated IR1 inversions in the presence of different divalent cations (bar graph). Plasmid pTH13170 (1 nM) was incubated with PsrA (125 nM) at 37ºC for 1 h. IR1 inversions were detected by PCR assays (agarose gel) and by qPCR assays (bar graph) using the P1/P3 primers (see Figure 8A). **(B)** Relative frequency of PsrA-mediated IR1 inversions using plasmid pTH13170 and the indicated concentrations of MgCl_2_ (bar graph).

**Figure S8. Binding reactions in the presence of divalent cations.** A non-labelled DNA fragment (10 nM) that contains the IR1.2 repeat was incubated with the indicated concentrations of PsrA in the absence of cations **(A)**, in the presence of 5 mM MgCl_2_ **(B)** or in the presence of 0.5 mM FeCl_2_ **(C)**. Binding reactions were loaded onto native PAA (6%) gels.

**Figure S9. Effect of PsrA concentration on the frequency of IR1 inversions.** Plasmids pTH13166 (1 nM) or pTH13170 (1 nM) (see Figure 8A) were incubated with different concentrations of PsrA at 37ºC for 1 h. Reaction mixtures contained 125 mM NaCl and 7.5 mM MgCl_2_. IR1 inversions were detected by PCR assays (agarose gels) and by qPCR assays (bar graphs).
